# Control of coordinatively unsaturated Zr sites in ZrO_2_ for efficient C–H bond activation

**DOI:** 10.1038/s41467-018-06174-5

**Published:** 2018-09-18

**Authors:** Yaoyuan Zhang, Yun Zhao, Tatiana Otroshchenko, Henrik Lund, Marga-Martina Pohl, Uwe Rodemerck, David Linke, Haijun Jiao, Guiyuan Jiang, Evgenii V. Kondratenko

**Affiliations:** 1 Leibniz-Institut für Katalyse e.V. an der Universität Rostock, Albert-Einstein-Straße 29a, Rostock, 18059 Germany; 2 State Key Laboratory of Heavy Oil Processing, China University of Petroleum Beijing, Beijing, 102249 China

## Abstract

Due to the complexity of heterogeneous catalysts, identification of active sites and the ways for their experimental design are not inherently straightforward but important for tailored catalyst preparation. The present study reveals the active sites for efficient C–H bond activation in C_1_–C_4_ alkanes over ZrO_2_ free of any metals or metal oxides usually catalysing this reaction. Quantum chemical calculations suggest that two Zr cations located at an oxygen vacancy are responsible for the homolytic C–H bond dissociation. This pathway differs from that reported for other metal oxides used for alkane activation, where metal cation and neighbouring lattice oxygen form the active site. The concentration of anion vacancies in ZrO_2_ can be controlled through adjusting the crystallite size. Accordingly designed ZrO_2_ shows industrially relevant activity and durability in non-oxidative propane dehydrogenation and performs superior to state-of-the-art catalysts possessing Pt, CrO_*x*_, GaO_*x*_ or VO_*x*_ species.

## Abstract (web summary)

Identifying active sites and designing rationally heterogeneous catalysts are not inherently straightforward due to their complexity. Here, the authors reveal the nature of active sites for efficient C–H bond activation in C1-C4 alkanes over bare ZrO_2_ and provide fundamentals for controlling their concentration.

## Introduction

The ability of scientists to elaborate and apply fundamental principles for rational design of heterogeneous catalysts is decisive for the global sustainable development and the environment protection applications. One possible way in this direction is to identify the active sites or key atomic structures^1,2^ and, for the most part, to use this knowledge for catalyst design and preparation. For example, owing to the well-defined structure (size and/or shape) and composition of nanoparticles of metals or metal oxides, they are successfully used for the development of supported catalysts with controlled properties^3–8^. Modifying structure of bulk metal oxides at a nanoscale level also has striking effects on their physico-chemical and catalytic properties^9–13^. The present study elucidates at molecular level the effects of nanostructure of bare ZrO_2_ on the activity and selectivity in the non-oxidative dehydrogenation of ethane, propane or iso-butane to the corresponding olefins.

In comparison with oil-based cracking technologies, which provide a major part of C_2_–C_4_ olefins for the chemical industry, ethane steam cracking and the dehydrogenation of propane or iso-butane are more environmentally friendly processes because they are based on natural/shale gas containing significantly less impurities than the oil-based feedstock. In addition, the dehydrogenation of propane (PDH) has been developed to close the gap between the demand and supply of propene^14,15^. This olefin is second to ethene as an important building block of the chemical industry with an annual production of about 80 million metric tons^15^. Contribution of PDH technology to the overall propene production will grow because new plants are under construction^16^. There are, however, flaws related to the commercial catalysts with supported CrO_*x*_ or Pt species^14,15^. According to the U.S. Occupational Safety and Health Administration^17^, workplace exposure to Cr(VI) may cause various health effects. A key drawback of Pt-based catalysts is their cost and deactivation triggered by sintering of Pt species. To re-disperse platinum, the catalysts are treated by ecologically harmful Cl_2_ or Cl-containing compounds^15^. Recently, we developed eco-friendly and cost-efficient catalysts on the basis of ZrO_2_, which had, however, to be promoted with metal oxides and contain supported Ru or Rh NP to show high activity in the non-oxidative dehydrogenation of propane, n-butane or iso-butane^18–20^. Coordinatively unsaturated Zr (Zr_cus_) and neighbouring lattice oxygen were suggested to participate in the dehydrogenation reaction. Yet, the promoter used to purposefully create Zr_cus_ sites either increased or decreased the activity hence proving the limitations of this classical approach for tailored catalyst design.

Here, we describe how nanostructuring of ZrO_2_ crystallites enables the identification of the nature of active sites for efficient C–H bond activation and the control of their concentration without the usage of any dopant or supported species. Acid–base, redox and electrical conductivity properties of ZrO_2_ can also be controlled through crystallite size. A structural model of the active site is established owing to our multidisciplinary approach combining density functional theory (DFT) calculations with a number of complementary experimental methods including catalytic tests. The active site consists of two Zr cations located at an oxygen vacancy, which homolytically break the C–H bond in alkanes. The kind of this site differs from that previously suggested by some of us for doped ZrO_2_-based catalysts^18,19^ and by other researchers for different metal oxides used for PDH^21–23^, where metal cation and neighbouring lattice oxygen were assumed to form the active site. Bare ZrO_2_ designed especially to maximize the concentration of the active sites shows industrially relevant performance in comparison with commercial-like catalysts containing CrO_*x*_ or Pt species and other alternative state-of-the-art catalysts.

## Results

### Controlling activity and selectivity of ZrO_2_

**Fig. 1:**
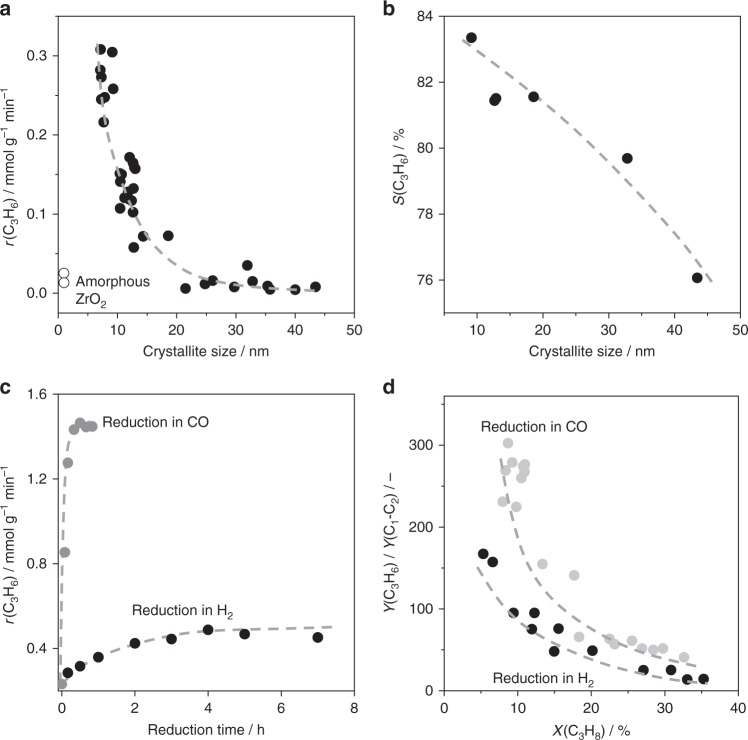
Catalytic property in PDH. **a** The rate of propene formation (*r*(C_3_H_6_)) versus the size of crystallites of differently prepared ZrO_2_ (Supplementary Table 1). **b** An integral selectivity to propene (*S*(C_3_H_6_)) calculated from the number of C_3_H_8_ moles converted and C_3_H_6_ moles formed within 1 h on C_3_H_8_ stream (Supplementary Fig. 4 and Supplementary Equation 2) over ZrO_2_ with different crystallite sizes. **c** *r*(C_3_H_6_) over ZrO_2_ after reduction in H_2_ or CO. **d** The effect of catalyst treatment by CO or H_2_ on the ratio of yields of propene to cracking products (*Y*(C_3_H_6_)/*Y*(C_1_–C_2_)). ZrO_2_ with the highest *S*(C_3_H_6_) in **b** was used in **c** and **d**. All these tests were performed at 550 °C using a feed containing 40 vol% C_3_H_8_ in N_2_

The scientific background for our study was the fact that the relative ratio of coordinatively unsaturated sites on the surface of metal oxides to their regular counterparts depends on the size of crystallites^24^. To check the influence of the nanostructure of bare ZrO_2_ for C–H bond activation in lower alkanes, we synthesized about 40 ZrO_2_ samples (Supplementary Table 1). Powder X-ray diffraction (XRD) analysis proved that they are mainly composed of the monoclinic phase (Supplementary Fig. 1) but differed in the average size of crystallites determined from the ($\bar 1$11) and (111) XRD reflexes. As seen in Fig. 1a, the rate of propene formation in PDH decreased by a factor of up to 70 with an increase in the size from 7 to 45 nm. There were no such strong differences between the catalysts in terms of the rate of propene formation if non-catalytic propane dehydrogenation played a significant role. The latter process is actually not relevant under the reaction conditions applied as proven by separate tests^19^ without catalysts.

It is also worth mentioning that for ZrO_2_ samples calcined at temperatures below 550 °C, the size of crystallites was determined after performing the PDH reaction (Supplementary Table 2). In addition to the crystallite size, long-range order of lattice oxygen and zirconium cations within at least a few nanometres in the crystal lattice of ZrO_2_ should be relevant for achieving high PDH activity. This conclusion is based on the fact that XRD-amorphous ZrO_2_ showed very low activity (Fig. 1a). The obtained relationship between the rate of propene formation and the size of crystallites is also valid when the rate is calculated with respect to catalyst surface area (Supplementary Fig. 2a). Thus, a simple effect of the crystallite size on the surface area and accordingly on the activity can be excluded, as the rate related to catalyst surface area increases with the area (Supplementary Fig. 2b-c).

No effect of the area would be visible if this catalyst property determined the activity exclusively. Moreover, although amorphous ZrO_2_ samples (ZrO_2__24 and ZrO_2__26 in Supplementary Table 1) possess the highest surface area, they showed the lowest activity. The PDH activity of ZrO_2_ is governed by the number of the active sites, which depends on the size of crystallites. A further experimental support for the importance of the latter catalyst property for PDH is the fact that apparent activation energy of propene formation depends on the size. The energy increases from about 140 to 300 kJ mol^−1^ with an increase in the size from 9 to 43 nm (Supplementary Fig. 3). This effect is related to the kind of catalytically active sites as will be demonstrated below when taking further catalytic and characterization studies as well as DFT calculations into account.

The activity-size relationship in Fig. 1a can be explained by an increase in the concentration of the catalytically active Zr_cus_ sites with a decrease in crystallite size. To validate the importance of such sites for propene formation, catalytic tests were performed with ZrO_2_ treated with either H_2_ or CO at 550 °C before testing in PDH at the same temperature. The idea was to create additional Zr_cus_ sites through removal of lattice oxygen from ZrO_2_ in form of H_2_O or CO_2_. The rate of propene formation increased by a factor of 2 after 6 h on H_2_ stream in comparison with the non-treated sample (Fig. 1c).

When CO was used as a reducing agent, a seven-time increase in the rate of propene formation was achieved after only 30 min treatment. Such distinctive effects of the reducing agents on the increase in the rate are related to their ability to remove lattice oxygen from ZrO_2_ as proven by temperature-programmed reduction (TPR) tests with H_2_ or CO (Supplementary Fig. 5). Our DFT calculations also predict that oxygen vacancies are easier formed through removal of lattice oxygen by CO than by H_2_ (Supplementary Table 3). The relevance of the so-generated surface defects for propene formation is further supported by the higher ratio of the yield of propene (*Y*(C_3_H_6_)) to that of cracking products (*Y*(C_1_–C_2_)) in a broad range of propane conversion obtained over the CO-treated catalyst in comparison to its H_2_-treated counterpart (Fig. 1d). This result suggests that propene and cracking products are formed on different sites.

**Fig. 2:**
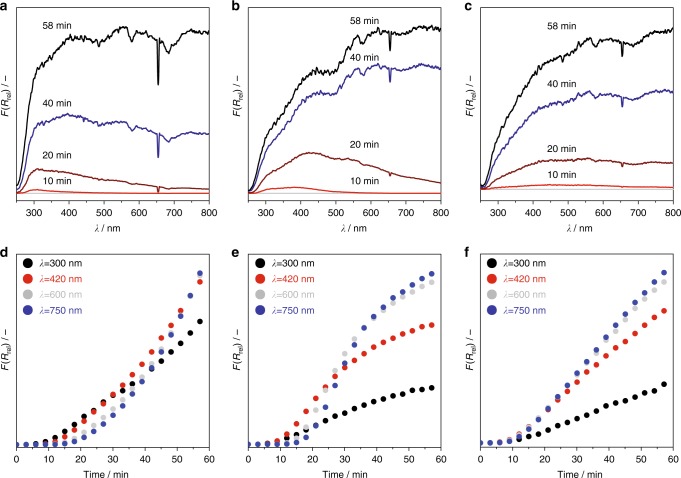
Coke formation in PDH. The UV-vis spectra of ZrO_2_ with **a** 9.1, **b** 13.0 or **c** 43.4 nm crystallites after different times on PDH stream. **d**–**f** Temporal evolution of Kubelka–Munk function at 300 (black circle), 420 (red circle), 600 (light grey circle) and 750 nm (blue circle) under PDH conditions. PDH was performed at 550 °C using a feed containing 40 vol% C_3_H_8_ in N_2_

The use of ZrO_2_ composed of small crystallites is also beneficial for propene selectivity, which increased with a decrease in the crystallite size (Fig. 1b). Such positive effect holds over a broad range of propane conversion (Supplementary Fig. 6). An insight into the formation and the kind of carbon deposits, which are the main undesired reaction product, was derived from the operando UV-vis analysis upon PDH over three ZrO_2_ samples with 9.1, 13.0 or 43.4 nm crystallites. The obtained UV-vis spectra expressed as the relative Kubelka–Munk function (*F*(*R*_rel_) in Eq. (2)) after different times on propane stream are shown in Fig. 2a–c. *F*(*R*_rel_) increased across the entire range of the UV-vis spectrum with rising time on propane stream. This increase must have occurred due to the deposition of coke species because the UV-vis spectra of H_2_-treated catalysts differ strongly (Supplementary Fig. 7). Absorption bands with the maxima at about 300, 420, 600 and 750 nm can be tentatively identified in the spectra of ZrO_2_ under PDH conditions. The latter two signals can be ascribed to polyaromatic graphitic species as previously suggested for PDH over a Cr-containing catalyst^25^. Temporal changes of the intensity of these bands under PDH conditions follow the same trend thus suggesting that they belong to the same carbon-containing species (Fig. 2d–f). The bands with the maxima in UV range (at 300 and 420 nm) should originate from low-condensed aromatic species, which differ from polyaromatic graphitic species also absorbing light above 500 nm. Different carbon-containing species are formed with different rates as seen in Fig. 2d–f showing temporal changes in the intensity of the corresponding bands with rising time of propane stream. When analysing the UV-vis spectra in Fig. 2a–c, it becomes obvious that the relative ratio of the intensity of absorption bands at 300 and 420 nm to that of the bands at higher wavelengths depends on the size of ZrO_2_ crystallites. The larger the size, the higher the fraction of polyaromatic graphitic species (absorption bands above 500 nm) is expected. In addition, there is an induction period before the UV-vis spectra start to change under the PDH conditions (Supplementary Fig. 8). Such delay may indicate that some coke precursors (seeds) must be initially formed before coke formation can proceed. Importantly, the duration of this induction period increased with a decrease in the size of ZrO_2_ crystallites (Supplementary Fig. 8).

On the basis of the above discussion, the effect of the size of ZrO_2_ crystallites on coke formation can be explained as follows. From a mechanistic point of view, adsorbed propene molecules interact with each other to initially form small aromatic structures followed by further oligomerization and condensation to large graphitic structures^15^. It is reasonable to suggest that propene molecules adsorbed horizontally to catalyst surface can recombine owing to their spatial location. Such adsorption should be more favourable for Zr sites located on flat ZrO_2_ surfaces in comparison to those located on corners or edges. Upon increasing the size of crystallites, the fraction of the former species will decrease and thus will result in a higher rate of carbon deposition. Exactly this trend was observed experimentally. Further experimental and theoretical studies are, however, required to clarify the size effect on coke formation.

**Fig. 3:**
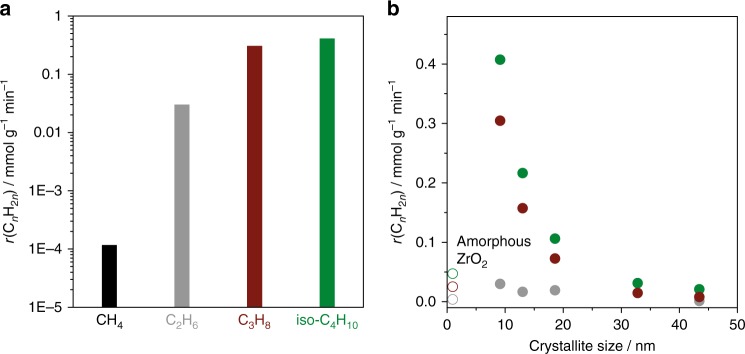
Catalytic activity for C–H bond breaking. The rate of olefin formation (*r*(C_*n*_H_2*n*_)), i.e. C_2_H_4_, C_2_H_4_, C_3_H_6_ and iso-C_4_H_8_ from CH_4_ (black), C_2_H_6_ (grey), C_3_H_8_ (dark red) and iso-C_4_H_10_ (green), respectively, **a** versus feed alkane over ZrO_2_ with the highest *S*(C_3_H_6_) in Fig. 1b or **b** versus the size of ZrO_2_ crystallites. The rate values obtained over amorphous ZrO_2_ in **b** are shown with open symbols. All these tests were performed using a feed containing 40 vol% alkane in N_2_ at 550 °C for C_2_H_6_ (grey bar in **a** and grey circle in **b**), C_3_H_8_ (dark red bar in **a** and dark red circles in **b**), and iso-C_4_H_10_ (green bar in **a** and green circle in **b**) or at 800 °C for  CH_4_ (black bar in **a**)

We turn our discussion back to catalyst activity to answer two important questions. Can ZrO_2_ activate C–H bond in other alkanes? Is the size–activity relationship valid for PDH only? To this end, we performed catalytic tests with iso-butane, ethane and methane with the latter possessing the highest C–H bond strength among alkanes. Due to the thermodynamic constrains, methane activation was investigated at 800 °C, while the tests with other alkanes were carried out at 550 °C, where PDH was also performed. Ethylene was the only product observed in the tests with methane. It is, however, worth mentioning that due to the low CH_4_ conversion of only 0.015%, it was difficult to precisely conclude if other products were also formed. Therefore, we do not discuss product selectivity in CH_4_ conversion tests. On the basis of previous studies on the oxidative coupling of methane^26^ and non-oxidative methane conversion to ethylene^27^, CH_3_ radical is suggested to be formed through breaking the C–H bond in CH_4_. Two such radicals recombine to C_2_H_6_ followed by its dehydrogenation to C_2_H_4_. ZrO_2_ is actually able to catalyse the latter reaction (Fig. 3a).

As seen in Fig. 3a, the feed alkanes can be ordered in terms of the rate of olefin formation as follows CH_4_ < C_2_H_6_ < C_3_H_8_ < iso-C_4_H_10_. This activity order actually correlates with the strength of the C–H bond in these alkanes. The corresponding values of the weakest C–H bond in these alkanes are 439.3, 420.5, 410.5 and 400.4 kJ mol^−1^. Regardless of the size of ZrO_2_ crystallites, the activity order did not change, while the dehydrogenation rate of C_2_H_6_, C_3_H_8_ and iso-C_4_H_10_ increased with a decrease in the size (Fig. 3b). CH_4_ conversion tests with ZrO_2_ materials differing in the size of their crystallites were not performed because this alkane requires too high temperature, where different structural changes in ZrO_2_ will occur.

### Benchmarking and demonstrating catalyst durability

**Fig. 4:**
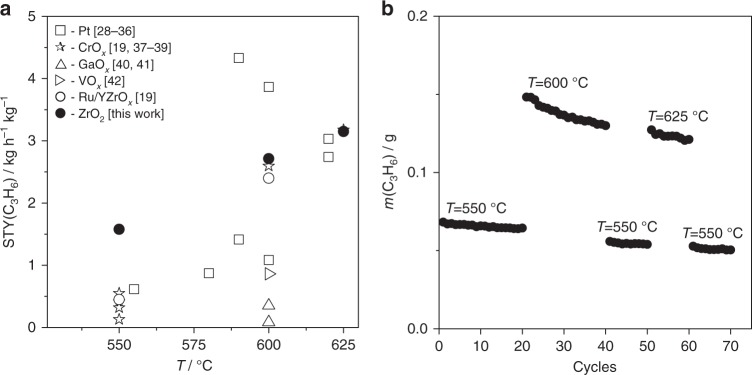
Comparison with literature data and durability. **a** Space time yield of propene (STY(C_3_H_6_)) obtained over ZrO_2_ and the most active catalysts from previous studies^28-42^ (Supplementary Table 3). The data at propane conversion above 25% were considered. **b** The amount of propene (*m*(C_3_H_6_)) formed within each 30 min PDH cycle in a series of 70 PDH/regeneration cycles at 550, 600 and 625 °C with WHSV of 1.57, 6.28 and 9.42 h^−1^, respectively. ZrO_2_ is the catalyst with the highest *S*(C_3_H_6_) in Fig. 1b

To demonstrate the potential of ZrO_2_ for PDH from an applied viewpoint, we determined the space time yield of propene (STY(C_3_H_6_)) over the best performing catalyst from Fig. 1b at different temperatures and propane conversion above 25%. The selectivity to propene was above 85%. Carbon deposits were the main side product with the selectivity of about 10%. Although they are not desired, such deposits indirectly contribute to minimizing overall costs in the course of CATOFIN process through using the heat released upon their combustion during catalyst regeneration phase for the endothermic PDH reaction^14^. Figure 4a shows a comparison of the present STY(C_3_H_6_) values with those obtained over state-of-the-art catalysts^28-42^ (Supplementary Table 4). All traditional catalysts with supported species of different metal oxides including chromium are less active than ZrO_2_. The latter also showed higher activity in comparison with doped ZrO_2_-based catalysts possessing supported Ru. Even in comparison with highly active but expensive Pt-based catalysts, ZrO_2_ proved its outstanding PDH activity.

Further practical relevance of ZrO_2_ was demonstrated over about 13 days on stream in a series of 70 PDH/regeneration cycles at 550, 600 and 625 °C. No reductive treatment was performed before PDH cycles because the catalyst can generate Zr_cus_ sites in situ through removal of lattice oxygen by propane/propene. The yield of propene within one PDH cycle initially increased and then decreased with rising time on stream at 550 °C but continuously decreased at higher temperatures (Supplementary Fig. 9). The increase is related to the formation of Zr_cus_ sites, while the decrease is due to catalyst deactivation caused by formation of carbon deposits, which could be removed upon oxidative catalyst regeneration as proven by stable catalyst performance within first 20 cycles at 550 °C (Fig. 4b).

When performing PDH at higher temperatures, the catalyst was less durable but showed high productivity. Such change of catalyst durability is due to increasing the size of crystallites caused by temperature-induced sintering process (Supplementary Fig. 10). Owing to small changes in the size, the catalyst was still active and durable at 550 °C (last 10 cycles in Fig. 4b) after the preceding PDH tests at 600 and 625 °C. Propene selectivity was about 90% (Supplementary Fig. 11).

### Role of ZrO_2_ nanostructure for formation of oxygen defects

**Fig. 5:**
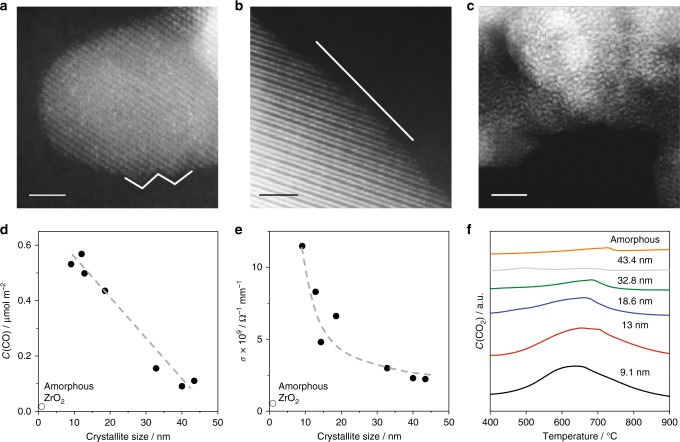
Morphology, acidic, conductivity and redox properties. HAADF-STEM images of ZrO_2_ with crystallites of **a** 9.1 nm, **b** 43.4 nm or **c** amorphous ZrO_2_. **d** Surface-normalized concentration of CO (*C*(CO)) determined from the amount of CO desorbed in CO-TPD. **e** Electrical conductivity at 550 °C in air. **f** Concentration of CO_2_ (*C*(CO_2_)) detected upon CO-TPR. Scale bars: 2 nm

High-angle annular dark-field scanning transmission electron microscopy (HAADF-STEM) was applied to characterize crystal morphology as a function of the size of crystallites. Figure 5a–c shows representative HAADF-STEM images of two crystalline samples with crystallites of 9.1 or 43.4 nm and one amorphous sample. Further representative images are given in Supplementary Fig. 12. There are significant differences between the samples in the structure of the surface depending on the crystallite size. Zigzag edges were seen in some images of the sample with 9.1 nm crystallites (Fig. 5a) and indicate the presence of a large number of corner atoms. Such surface defects should be coordinatively unsaturated zirconium and/or oxygen ions. They were not observed in the sample with 43.4 nm crystallites. Nearly perfect lattice planes with no corner atoms are typical for this sample (Fig. 5b), while no lattice planes are visible for the XRD-amorphous sample (Fig. 5c).

The HAADF-STEM technique can provide only limited quantitative information about the concentration and distribution of defect sites. In this regard, we applied surface-sensitive temperature-programmed desorption (TPD) of probe molecules differing in their Lewis basicity strength. For minimizing the effect of Brønsted sites on adsorption/desorption properties of ZrO_2_^20^, all TPD tests were carried out with the samples treated at 550 °C to remove acidic OH groups before adsorbing probe molecules.

CO naturally adsorbing on the Zr_cus_ sites (Lewis acidic sites) desorbed between 250 and 450 °C (Supplementary Fig. 13). The surface-normalized concentration of sites for CO adsorption was established to increase with a decrease in the size of crystallites (Fig. 5d), while the temperature of maximal rate of CO desorption was not affected (Supplementary Fig. 13). The lowest amount was detected for amorphous ZrO_2_. A similar effect of the crystallite size on the concentration of adsorption sites for NH_3_ and C_3_H_6_ was also obtained when these compounds were used as probe molecules for titrating Lewis acidic sites (Supplementary Fig. 14). Regardless of the probe molecule the rate of alkene formation from C_2_H_6_, C_3_H_8_ and iso-C_4_H_10_ rose with an increase in the concentration of the accordingly determined Lewis acidic sites (Supplementary Fig. 15). Thus, the TPD results suggest that nanostructure is vital for the stabilization/formation of surface Lewis acidic defects.

We also applied electrical conductivity tests at 550 °C for semi-quantitative analysis of the number of bulk anion vacancies, which are responsible for oxygen-ionic conductivity in ZrO_2_-based materials^43^ and direct indicators for the presence of Zr_cus_ sites. To check the contribution of electronic conduction, we analysed the effect of oxygen partial pressure on overall conductivity. Regardless of the size of crystallites, the conductivity decreased by only a factor of less than 2, which is lower than 21 as expected for a pure p-conductor upon reducing the pressure from 20 kPa to about 10^−4^ kPa (Supplementary Table 5). This result proves that all tested ZrO_2_ samples mainly possess ionic conductivity. As seen in Fig. 5e the conductivity increased with a decrease in the size of crystallites. Such relationship is due to the higher concentration of anion vacancies or/and higher  diffusivity of O^2^^−^ in the samples with smaller crystallite size. It is worth mentioning that the conductivity of amorphous ZrO_2_ was very low (Fig. 5e). Thus, an optimal size of ZrO_2_ crystallites is required to achieve the highest conductivity.

The crystallite size is also decisive for the formation of anion vacancies as proven by TPR tests with CO (Fig. 5f). The smaller the size, the higher the amount of lattice oxygen was removed in form of CO_2_. This result is in agreement with previous DFT calculations predicting that the energy for the formation of an oxygen vacancy in ZrO_2_ lowers for nanoscale particles^44^. However, amorphous ZrO_2_ produced very low amount of CO_2_ from CO (Fig. 5f). Thus, there should be a minimal size of crystallites, below which the removal of lattice oxygen from ZrO_2_ will be hindered.

### Molecular level details on C_3_H_8_ dehydrogenation over ZrO_2_

**Fig. 6:**
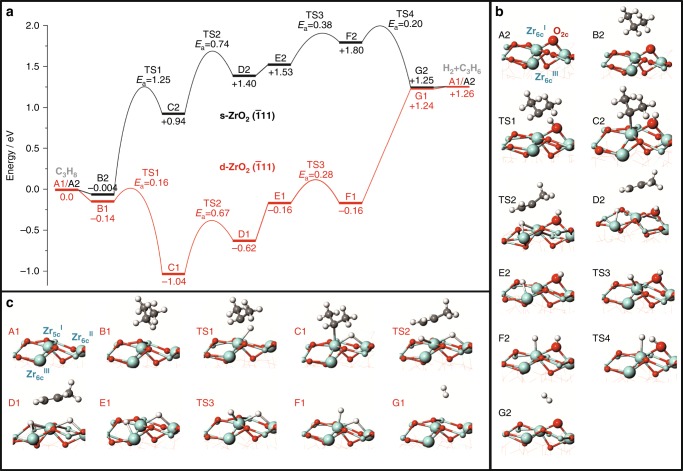
Mechanism of PDH. **a** The calculated energy profiles along the pathways of propane dehydrogenation to propene and the optimized structures of intermediates and transition states (TS) on **b** s-ZrO_2_($\bar 1$11) and **c** d-ZrO_2_($\bar 1$11) surfaces (Cyan, grey, red and white symbols stand for Zr, C, O and H, respectively)

With the purpose to derive molecular insights into the kind of the catalytically active sites for propane dehydrogenation to propene over monoclinic ZrO_2_, we performed DFT calculations. The focus was put on the role of Zr_cus_ sites. As the ($\bar 1$11) facet represents the most stable surface plane of monoclinic ZrO_2_^45–47^, it was used as the basis for computing stoichiometric (s-ZrO_2_($\bar 1$11)) and oxygen-defective (d-ZrO_2_($\bar 1$11)) surfaces. Computational details are given in Supplementary Fig. 16, Supplementary Table 3 and Supplementary Table 6. Although the energies for activating C_3_H_8_ at the methylene or methyl C–H bond to yield *iso*-C_3_H_7_ or *n*-C_3_H_7_ are close (Supplementary Table 7), subtraction of a second hydrogen to yield propene is kinetically more favourable for the *iso*-C_3_H_7_ fragment. This result is in agreement with previous DFT calculations of PDH over other metal oxides^21–23^. Therefore, the first step of propane activation in our calculations was cleavage of the methylene C–H bond. The full potential energy surfaces of the most preferred routes on both surfaces are shown in Fig. 6, while the Gibbs free energy profiles are given in Supplementary Fig. 17. This figure shows that the apparent free energy barrier for propane dehydrogenation over the d- ZrO_2_($\bar 1$11) facet is 1.66 eV lower than that over the s-ZrO_2_($\bar 1$11) facet (2.52 vs. 4.18 eV) thus indicating that the dehydrogenation reaction is kinetically more favoured over the d-ZrO_2_($\bar 1$11) facet than over the s-ZrO_2_($\bar 1$11) facet.

Methylene C–H bond activation in propane: To identify the active sites and the modes of methylene C–H bond dissociation on the s-ZrO_2_($\bar 1$11) surface, we computed this route on a centre consisting either of one Zr site and one O site or two O sites. For the sake of completeness, six- and seven-fold coordinated Zr cations (Zr_6c_^I^, Zr_7c_^II^, Zr_6c_^III^ and Zr_6c_^IV^) as well as twofold and threefold coordinated oxygen anions (O_2c_, O_3c_^I^, O_3c_^II^, O_3c_^III^ and O_3c_^IV^) were considered (Supplementary Fig. 16). A Zr–O pair was found to be the most favourable centre to break the methylene C–H bond through a heterolytic route (Supplementary Fig. 18 and Supplementary Table 7). The Zr_6c_^I^–O_2c_ site should possess the highest reactivity because of the formation of the most stable Zr_6c_^I^–*iso*-C_3_H_7_ and O_2c_–H intermediates owing to the stronger basicity of O_2c_ in comparison with O_3c_. This step is endothermic by 0.94 eV and has an activation barrier of 1.25 eV (Fig. 6).

In contrast to s-ZrO_2_($\bar 1$11), methylene C–H bond cleavage over d-ZrO_2_($\bar 1$11) is a homolytic process without involving lattice oxygen. This reaction should occur on two adjacent Zr cations, *i.e*. Zr_5c_^I^ and Zr_6c_^II^ ([Zr_5c_^I^, Zr_6c_^II^]–O_v_) or Zr_5c_^I^ and Zr_6c_^III^ ([Zr_5c_^I^, Zr_6c_^III^]–O_v_), and is exothermic by 1.04 or 0.55 eV (Fig. 6 and Supplementary Table 8). The Zr_5c_^I^ and Zr_6c_^II^ cations are located at an anion vacancy (O_v_), while Zr_6c_^III^ is a regular surface site. The formed *iso*-C_3_H_7_ fragment is bound to the Zr_5c_^I^ site, while the H atom is located in a bridged position between Zr_5c_^I^ and Zr_6c_^II^ or between Zr_5c_^I^ and Zr_6c_^III^. On the basis of the activation barrier (0.16 vs. 0.73 eV) for breaking the methylene C–H bond (Supplementary Fig. 19), the [Zr_5c_^I^, Zr_6c_^II^]–O_v_ site should be more reactive than the [Zr_5c_^I^, Zr_6c_^III^]–O_v_ site. With respect to the Zr_6c_^I^–O_2c_ site on the s-ZrO_2_($\bar 1$11) surface, the activation barrier for this reaction pathway decreases by 1.09 eV in the presence of oxygen vacancy (Fig. 6) thus strongly facilitating propane activation.

To check the importance of oxygen defects for activation of methane, which is the most inert alkane, we computed breaking C–H bond over the s-ZrO_2_($\bar 1$11) and d-ZrO_2_($\bar 1$11) surfaces. Similarly to propane activation, Zr_6c_^I^–O_2c_ and [Zr_5c_^I^, Zr_6c_^II^]-O_v_ should be the corresponding active sites. The presence of oxygen defect was established to be essential for breaking the C–H bond in CH_4_, too. The activation barrier on the Zr_6c_^I^–O_2c_ site is 1.14 eV but is only 0.10 eV on the [Zr_5c_^I^, Zr_6c_^II^]-O_v_ site. Moreover, CH_4_ activation on the latter centre is exothermic by 1.06 eV.

C_3_H_6_ and H_2_ formation: After having identified the first step of propane activation over s-ZrO_2_($\bar 1$11), we examined transformation routes of the Zr_6c_^I^–*iso*-C_3_H_7_ and H–O_2c_ intermediates. The most thermodynamically and kinetically preferred way for Zr_6c_^I^–*iso*-C_3_H_7_ conversion is the cleavage of the methyl C–H bond at the Zr_6c_^III^ site (Supplementary Fig. 20). It is an endothermic process (0.46 eV) and has an activation barrier of 0.74 eV (C2 to D2 in Fig. 6), which is 0.51 eV lower than that for the activation of the first methylene C–H bond in propane. The so-formed propene adsorbs weakly (0.13 eV) at the Zr_6c_^I^ site, while the co-generated H fragment is bound to the neighbouring Zr_6c_^III^ site (Fig. 6).

When one H atom is subtracted from the methyl group of Zr_5c_^I^–*iso*-C_3_H_7_ stabilized on the d-ZrO_2_($\bar 1$11) surface (Fig. 6 and Supplementary Fig. 21), weakly adsorbed propene (0.46 eV) is formed (TS2, Fig. 6). The remaining H atom is bound to the Zr_5c_^I^ and Zr_6c_^III^ cations. This process is endothermic by 0.42 eV and has an activation barrier of 0.67 eV, which is slightly lower than that (0.74 eV) required for the activation of Zr_6c_^I^–*iso*-C_3_H_7_ over the stoichiometric surface. In addition to the mechanism and the kinetics of propene formation, the presence of anion vacancies also affects H_2_ formation (Fig. 6). This process on the s-ZrO_2_ ($\bar 1$11) surface has an effective activation barrier of 0.47 eV and is exothermic by 0.28 eV, while the formation of H_2_ on the d-ZrO_2_ ($\bar 1$11) surface is endothermic by 1.40 eV (H_2_ dissociation is barrierless).

## Discussion

The work presented here explains at molecular level the importance of oxygen defects on the surface of ZrO_2_ for efficient C–H bond activation in C_1_–C_4_ alkanes. Owing to the presence of such defects, this typically non-reducible metal oxide showed unexpectedly high activity in the non-oxidative propane dehydrogenation to propene and outperformed the state-of-the-art catalysts possessing supported metal oxides or platinum. Its unique performance is related to the presence of surface coordinatively unsaturated Zr cations (Zr_cus_) located at anion vacancies in the lattice of ZrO_2_. Our DFT calculations predict that Zr_cus_ sites open an alternative pathway of propene and hydrogen formation with a lower activation barrier in comparison with non-defect ZrO_2_. Two Zr_cus_ sites directly participate in homolitic C–H bond activation, while lattice oxygen and zirconium cation are required for this process on the stoichiometric surface of ZrO_2_. Although the concentration of Zr_cus_ can be adjusted in a controlled manner through the size of ZrO_2_ crystallites, further improvements are expected when the coordination number of zirconium cations, their location on certain facets and/or the shape of crystallites can also be tuned. Our approach presented herein can be extended to other bulk oxides of non-reducible metals and offers the opportunity for tailoring their acid–base and redox properties as well as electrical conductivity for specific applications.

## Methods

### Catalyst preparation

ZrO_2_ samples were prepared by hydrothermal, precipitation, sol-gel, hard-template methods or simple decomposition of different zirconium salts. The used chemicals and selected physico-chemical properties of the synthesized materials are given in Supplementary Table 1. Further preparation details are provided in Supplementary Note 1.

### Catalyst characterization

XRD powder patterns were recorded on a Panalytical X’Pert diffractometer equipped with a Xcelerator detector, automatic divergence slits and Cu tube (kα1/α2 radiation, 40 kV, 40 mA, *λ* = 0.015406 nm, 0.0154443 nm). Cu beta-radiation was excluded by using nickel filter foil. The measurements were performed in 0.0167° steps and 25 s of data collecting time per step. Peak positions and profile were fitted with Pseudo-Voigt function using the HighScore Plus software package (Panalytical). The PDF-2 database of the International Centre of Diffraction Data (ICDD) was used for phase identification. The integral breadth method using the Scherrer equation under the assumption of spherically shaped crystallites was applied for calculating crystallite size from the ($\bar 1$11) and (111) reflection peak and the average values are reported. The K parameter was set to 1.0747. The adjustment of the used diffractometer is weekly checked according to the Si (111) reflection peak at 28.433°. The deviation of the peak position is within the standard deviation (0.004°). We have also measured XRD of five randomly taken probes of ZrO_2_ with an estimated crystallite size of 9.1 nm, which is one of the lowest values. The size of crystallites was determined with an error of 0.02 nm. Thus, the error in determining the size of crystallites is below 1%.

Transmission electron microscopy measurements were carried out at 200 kV with an aberration-corrected JEM-ARM200F (JEOL, Corrector: CEOS). The aberration-corrected STEM imaging (High-Angle Annular Dark Field) was performed with a spot size of approximately 0.1 nm, a convergence angle of 30–36^o^ and collection semi-angle of 90–170 mrad.

Acid–base, redox and electrical properties of selected samples were determined from temperature-programmed desorption tests using CO, NH_3_ or C_3_H_6_ as probe molecules, temperature-programmed reduction tests with H_2_ or CO and electrical conductivity measurements respectively. Technical details of these tests are given in Supplementary Note 2.

In situ UV-vis tests were performed at 550 °C in an in-house developed set-up equipped with an AVASPEC fibre optical spectrometer (Avantes), a DH-2000 deuterium-halogen light source and a CCD array detector. A high-temperature reflection UV-vis probe consisting of six radiating optical fibres and one reading fibre was threaded through the furnace to face quartz reactor walls at the position where catalysts were held^48^. A feed containing 40 vol% C_3_H_8_ in N_2_ was used in these tests. Initial propane conversion was about 10%.

For analysing carbon deposition during PDH, we defined relative reflectance (*R*_rel_) as given in Eq. (1) as the ratio of the reflectance of the catalysts with coke deposits (*R*_DH_) to the fully oxidized ones (*R*_O2_). From this reflectance, we calculated the relative Kubelka–Munk function *F*(*R*_rel_) according to Eq. (2).

1 $$R_{{\mathrm{rel}}} = \frac{{R_{{\mathrm{DH}}}}}{{R_{{\mathrm{O}}_2}}}$$

2 $$F\left( {R_{{\mathrm{rel}}}} \right) = \frac{{(1 - R_{{\mathrm{rel}}})^2}}{{2 \times R_{{\mathrm{rel}}}}}$$

### Catalytic tests

Catalytic tests with C_2_H_6_, C_3_H_8_ and iso-C_4_H_10_ were performed at 1 bar between 550 and 625 °C using an in-house developed set-up consisting of 15 continuous-flow fixed-bed quartz tubular (length and inner diameter are 465 and 4 mm, respectively) reactors operating in parallel. Tests with CH_4_ were carried out at 800 °C in an in-house developed set-up consisting of six continuous-flow fixed-bed quartz tubular (length and inner diameter are 330 and 4 mm respectively) reactors operating in parallel. Depending on specific purposes, catalytic tests were performed with oxidized (treated in air at the reaction temperature) or reduced (treated in either CO/N_2_ = 57/43 or H_2_/N_2_ = 57/43 at the reaction temperature) catalysts. Feeds containing 40 vol% C_2_H_6_, 40 vol% C_3_H_8_, 40 vol% iso-C_4_H_10_ or 40 vol% CH_4_ in N_2_ were used. The feed components and the reaction products were analysed by an on-line gas chromatograph (Agilent 6890) equipped with PLOT/Q (for CO_2_), AL/S (for hydrocarbons) and Molsieve 5 (for H_2_, O_2_, N_2_ and CO) columns as well as flame ionization and thermal conductivity detectors. Further details about catalytic tests as well as formulae for calculating various catalyst characteristics are provided in Supplementary Note 3.

### DFT calculations

Spin-polarized and periodic density functional theory (DFT) calculations were carried out by using the Vienna ab initio simulation package (VASP)^49,50^. Exchange and correlation were treated within the Perdew–Burke–Ernzerhof generalized gradient approximation (GGA-PBE)^51^. To obtain accurate energies with errors of less than 1 meV per atom, a cut-off energy of 400 eV was used. Geometry optimization was converged until forces acting on atoms were lower than 0.02 eV/Å, whereas the energy threshold defining self-consistency of the electron density was set to 10^−4^ eV. The climbing image nudged elastic band (CI-NEB) method with eight images was applied for finding transition states and minimum energy paths of all reactions^52^. The final transition state structures were refined by using the quasi-Newton algorithm until the Hellman–Feynman forces on each ion were lower than 0.02 eV/Å. The normal mode frequency analysis was performed to validate the optimized transition states and each authentic transition state has only one imaginary frequency along the reaction coordinates. The resulted zero-point vibrational energies (ZPE) from the frequency analysis are included in our energetic comparison and discussion. For the optimization of the bulk structure, the lattice parameters of the monoclinic ZrO_2_ (*m*-ZrO_2_) phase were determined by minimizing the total energy of the unit cell by using a conjugated gradient algorithm to relax the ions. A 7×7×7 Monkhorst−Pack k point grid was used for sampling the Brillouin zone^53^. Additional details are provided in Supplementary Methods. In addition, we also tested the corrections of Hubbard term (PBE + U) and dispersion (DFT + D3). These new data are now presented in Supplementary Fig. 22 and Supplementary Fig. 23.

## Electronic supplementary material

Supplementary Information

Peer Review File

## Data Availability

The authors declare that the data supporting the findings of this study are available within the paper and its supplementary information. Further information is also available from the corresponding authors upon reasonable request.

## References

[1] Nørskov JK, Bligaard T, Rossmeisl J, Christensen CH (2009). Towards the computational design of solid catalysts. Nat. Chem..

[2] Buurmans ILC, Weckhuysen BM (2012). Heterogeneities of individual catalyst particles in space and time as monitored by spectroscopy. Nat. Chem..

[3] Zaera F (2013). Nanostructured materials for applications in heterogeneous catalysis. Chem. Soc. Rev..

[4] Munnik P, de Jongh PE, de Jong KP (2015). Recent developments in the synthesis of supported catalysts. Chem. Rev..

[5] Zečević J, Vanbutsele G, de Jong KP, Martens JA (2015). Nanoscale intimacy in bifunctional catalysts for selective conversion of hydrocarbons. Nature.

[6] Jagadeesh RV (2017). MOF-derived cobalt nanoparticles catalyze a general synthesis of amines. Science.

[7] Shan J (2017). Mild oxidation of methane to methanol or acetic acid on supported isolated rhodium catalysts. Nature.

[8] Marcinkowski MD (2018). Pt/Cu single-atom alloys as coke-resistant catalysts for efficient C–H activation. Nat. Chem..

[9] Wu HB, Chen JS, Hng HH, Lou XW (2012). Nanostructured metal oxide-based materials as advanced anodes for lithium-ion batteries. Nanoscale.

[10] Mahato RN (2013). Ultrahigh magnetoresistance at room temperature in molecular wires. Science.

[11] Zhang Z (2015). Ultrathin inorganic molecular nanowire based on polyoxometalates. Nat. Commun..

[12] Zhang Z (2016). Acidic ultrafine tungsten oxide molecular wires for cellulosic biomass conversion. Angew. Chem. Int. Ed..

[13] Trovarelli A, Llorca J (2017). Ceria catalysts at nanoscale: how do crystal shapes shape catalysis?. ACS Catal..

[14] Vora BV (2012). Development of dehydrogenation catalysts and processes. Top. Catal..

[15] Sattler JJHB, Ruiz-Martinez J, Santillan-Jimenez E, Weckhuysen BM (2014). Catalytic dehydrogenation of light alkanes on metals and metal oxides. Chem. Rev..

[16] Boswell, C. On-purpose technologies ready to fill propylene gap. https://www.icis.com/resources/news/2012/04/16/9549968/on-purpose-technologies-ready-to-fill-propylene-gap/ (2012).

[17] Occupational Safety and Health Administration. Hexavalent chromium. https://www.osha.gov/SLTC/hexavalentchromium/index.html (accessed 2 Sept 2018).

[18] Otroshchenko T (2015). ZrO_2_-based alternatives to conventional propane dehydrogenation catalysts: active sites, design and performance. Angew. Chem. Int. Ed..

[19] Otroshchenko T (2017). ZrO_2_-based unconventional catalysts for non-oxidative propane dehydrogenation: factors determining catalytic activity. J. Catal..

[20] Otroshchenko TP (2017). Non-oxidative dehydrogenation of propane, n-butane, and isobutane over bulk ZrO_2_-based catalysts: effect of dopant on the active site and pathways of product formation. Catal. Sci. Tech..

[21] Liu Y, Li ZH, Lu J, Fan KN (2008). Periodic density functional theory study of propane dehydrogenation over perfect Ga_2_O_3_(100) surface. J. Phys. Chem. C..

[22] Fu H (2006). Periodic density functional theory study of propane oxidative dehydrogenation over V_2_O_5_(001) surface. J. Am. Chem. Soc..

[23] Rozanska X, Fortrie R, Sauer J (2014). Size-dependent catalytic activity of supported vanadium oxide species: oxidative dehydrogenation of propane. J. Am. Chem. Soc..

[24] Rodriguez JA, Fernández-García M (2007). Synthesis, Properties, and Applications of Oxide Nanomaterials.

[25] Nijhuis TA, Tinnemans SJ, Visser T, Weckhuysen BM (2004). Towards real-time spectroscopic process control for the dehydrogenation of propane over supported cromium oxide catalysts. Chem. Eng. Sci..

[26] Kondratenko, E. V. & Baerns, M. *Handbook of Heterogeneous Catalysis* 2nd Edn, 3010–3023 (Wiley‐VCH, Weinheim, 2008).

[27] Guo X (2014). Direct, nonoxidative conversion of methane to ethylene, aromatics, and hydrogen. Science.

[28] Sattler JJHB (2014). Platinum-promoted g-/Al_2_O_3_ as highly active, selective, and stable catalyst for the dehydrogenation of propane. Angew. Chem. Int. Ed..

[29] De Cola PL, Gläser R, Weitkamp J (2006). Non-oxidative propane dehydrogenation over Pt–Zn-containing zeolites. Appl. Catal. A.

[30] Xiong H (2017). Thermally stable and regenerable platinum–tin clusters for propane dehydrogenation prepared by atom trapping on ceria. Angew. Chem. Int. Ed..

[31] Li J (2017). Size effect of TS-1 supports on the catalytic performance of PtSn/TS-1 catalysts for propane dehydrogenation. J. Catal..

[32] Pham HN, Sattler JJHB, Weckhuysen BM, Datye AK (2016). Role of Sn in the regeneration of Pt/γ-Al_2_O_3_ light alkane dehydrogenation catalysts. ACS Catal..

[33] Im J, Choi M (2016). Physicochemical stabilization of Pt against sintering for a dehydrogenation catalyst with high activity, selectivity, and durability. ACS Catal..

[34] Shi L (2015). Al_2_O_3_ nanosheets rich in pentacoordinate Al^3+^ ions stabilize Pt-Sn clusters for propane dehydrogenation. Angew. Chem., Int. Ed..

[35] Zhu H (2014). Sn surface-enriched Pt–Sn bimetallic nanoparticles as a selective and stable catalyst for propane dehydrogenation. J. Catal..

[36] Jiang F (2015). Propane dehydrogenation over Pt/TiO_2_–Al_2_O_3_ catalysts. ACS Catal..

[37] Alcántara-Rodríguez M, Rodríguez-Castellón E, Jiménez-López A (1999). Propane dehydrogenation on mixed Ga/Cr oxide pillared zirconium phosphate materials. Langmuir.

[38] Zhang X, Yue Y, Gao Z (2002). Chromium oxide supported on mesoporous SBA-15 as propane dehydrogenation and oxidative dehydrogenation catalysts. Catal. Lett..

[39] Michorczyk P, Pietrzyk P, Ogonowski J (2012). Preparation and characterization of SBA-1–supported chromium oxide catalysts for CO_2_ assisted dehydrogenation of propane. Micro. Mesopor. Mater..

[40] Michorczyk P, Ogonowski J (2003). Dehydrogenation of propane to propene over gallium oxide in the presence of CO_2_. Appl. Catal. A.

[41] Xu BJ (2006). Support effect in dehydrogenation of propane in the presence of CO_2_ over supported gallium oxide catalysts. J. Catal..

[42] Liu G (2016). Nature of the active sites of VO_*x*_/Al_2_O_3_ catalysts for propane dehydrogenation. ACS Catal..

[43] Etsell TH, Flengas SN (1970). Electrical properties of solid oxide electrolytes. Chem. Rev..

[44] Ruiz Puigdollers A, Schlexer P, Tosoni S, Pacchioni G (2017). Increasing oxide reducibility: the role of metal/oxide interfaces in the formation of oxygen vacancies. ACS Catal..

[45] Christensen A, Carter EA (1998). First-principles study of the surfaces of zirconia. Phys. Rev. B.

[46] Piskorz W (2011). Periodic DFT and atomistic thermodynamic modeling of the surface hydration equilibria and morphology of monoclinic ZrO_2_ nanocrystals. J. Phys. Chem. C..

[47] Kouva S, Honkal K, Lefferts L, Kanervo J (2015). Review: monoclinic zirconia, its surface sites and their interaction with carbon monoxide. Catal. Sci. Tech..

[48] Kondratenko EV (2010). Using time-resolved methods to monitor and understand catalytic oxidation reactions. Catal. Today.

[49] Kresse G, Furthmüller J (1996). Efficiency of ab-initio total energy calculations for metals and semiconductors using a plane-wave basis set. Comput. Mater. Sci..

[50] Kresse G, Furthmüller J (1996). Efficient iterative schemes for ab initio total-energy calculations using a plane-wave basis set. Phys. Rev. B.

[51] Perdew JP, Burke K, Ernzerhof M (1996). Generalized gradient approximation made simple. Phys. Rev. Lett..

[52] Henkelman G, Uberuaga BP, Jónsson H (2000). A climbing image nudged elastic band method for finding saddle points and minimum energy paths. J. Chem. Phys..

[53] Monkhorst HJ, Pack JD (1976). Special points for Brillouin-zone integrations. Phys. Rev. B.

