# Supplementary material for: Control of coordinatively unsaturated Zr sites in ZrO2 for efficient C–H bond activation
Source: Nat Commun. 2018 Sep 18;9:3794. doi: 10.1038/s41467-018-06174-5 (PMC6143600; doi:10.1038/s41467-018-06174-5)
Supplement: Supplementary file 1 — Supplementary Information [file 41467_2018_6174_MOESM1_ESM.pdf]

Supplementary Information

Control of coordinatively unsaturated Zr sites in ZrO_2 for efficient C-H bond activation

Zhang et al.

Supplementary Note 1

Supplementary Table 1 provides preparation details and selected physical properties of all ZrO_2 catalysts. The catalysts from ZrO_2_1 to ZrO_2_23 were prepared by hydrothermal method according to Ref.[1]. For the synthesis of the catalysts ZrO_2_1 to ZrO_2_7, 12.748 g of $ZrO(NO_3)_2 \cdot xH_2O$ (99%, Aldrich) were dissolved in 30 mL of H_2O. Separately, 21.622 g of urea were dissolved in 30 mL of H_2O. The resulting solutions were mixed together to obtain a concentration of Zr^{4+} of 0.6 $mol \cdot L^{-1}$. The solution was then transferred into a Teflon lined stainless-steel autoclave. Crystallization was made at 180 °C under autogenous pressure for 20 h. Hereafter, the precipitate was washed thoroughly with deionized water and dried at 110 °C overnight. The obtained powder was calcined at 110, 200, 250, 300, 350, 400 or 450 °C for 4 h. The resulting catalysts were denoted as ZrO_2_1, ZrO_2_2, ZrO_2_3, ZrO_2_4, ZrO_2_5, ZrO_2_6, ZrO_2_7 respectively. The catalysts ZrO_2_8 to ZrO_2_14 were prepared in a similar way. Briefly, 5.312 g of $ZrO(NO_3)_2 \cdot xH_2O$ were dissolved in 7.5 mL of H_2O, 1.74 g of P123 (Aldrich, average M_n ~ 5800) were dissolved in 37.5 mL of H_2O, 18.018 g of urea were dissolved in 17.5 mL of H_2O. The above solutions were mixed together to obtain a concentration of Zr^{4+} of 0.24 $mol \cdot L^{-1}$. For the preparation of the catalysts ZrO_2_13 and ZrO_2_14, the solution was aged at 100 °C for 2 days. For the synthesis of other catalysts, the aging temperature was kept at 80 °C. Hereafter, the solution was transferred into autoclave. Hydrothermal treatment was performed at 180 °C for all catalysts. Crystallization time was 6, 12, 24, 48, 72, 24, and 48 h for ZrO_2_8, ZrO_2_9, ZrO_2_10, ZrO_2_11, ZrO_2_12, ZrO_2_13, ZrO_2_14 respectively. After that, all the catalysts were dried at 110 ^{o}C overnight and then calcined at 550 °C for 4 h. The catalysts from ZrO_2_15 to ZrO_2_21 were prepared in a similar way as ZrO_2_11, but in the presence of F127 (Aldrich), P123, Diethylenetriamine (99%, Aldrich), Sodium dodecyl sulphate (>98.5%, Aldrich), CTAB (>98%,

Aldrich), Dodecylamine (98%, Aldrich) and NH_4F (>98%, Fluka) with the molar ratio of such additives to Zr^{4+} of 0.1, 0.1, 0.5, 0.1, 0.5, 0.5, and 0.3 respectively. The synthesis procedure of ZrO_2_22 was similar to that of ZrO_2_15. The only difference was the absence of the template P123. ZrO_2_23 was prepared similarly as ZrO_2_21, but the NH_4F to Zr^{4+} molar ratio was 1.4, and the solution was crystallized at 105 °C for 24 h.

ZrO_2_24 was prepared using $ZrO(NO_3)_2{\cdot}xH_2O$ as a precursor. The procedure was as follows. 7.622 g of $ZrO(NO_3)_2{\cdot}xH_2O$ was firstly dissolved in 500 mL of H_2O. Then, ethylenediamine was added dropwise to the solution until pH reached 11. Hereafter, the solution was stirred for 30 min at room temperature, heated up to 100 °C and stirred for 4 days. The obtained precipitate was filtered, dried and calcined at 550 °C for 4 h.

ZrO_2_25 was prepared using $ZrOCl_2{\cdot}8H_2O$ (>95%, Fluka) as a precursor. Briefly, required amount of $ZrOCl_2{\cdot}8H_2O$ was dissolved in deionized water to obtain solution with a concentration of Zr^{4+} of 1.0 $mol{\cdot}L^{-1}$. An aqueous solution of ammonia was then added dropwise under stirring until pH reached 9. The precipitate formed was aged overnight, filtered and washed several times with deionized water until no more chloride ions were identified in filtrate (reaction with $AgNO_3$). The solid was dried at 110 °C overnight and calcined at 550 °C for 4 h. To synthesize ZrO_2_26, approximately 0.35 M aqueous solution of $ZrOCl_2{\cdot}8H_2O$ was firstly prepared. This solution was added dropwise under stirring to 5 M aqueous solution of ammonia with 150% in excess. Formed white precipitate was digested in mother liquor at 100 °C under open reflux for 192 h. Then, the solid was filtrated, washed with deionized water until no more chloride ions were detected in filtrate, dried at 110 °C overnight and calcined at 550 °C for 4 h.

The catalysts ZrO_2_27 - ZrO_2_30 were prepared using zirconium(IV) butoxide solution (80 wt% in 1-butanol, Aldrich) as a precursor and ethanol as a solvent. Typically, 300 mL of ethanol were mixed with 1.0 mL of H_2O. Hereafter, 9.592 g of $Zr(OBu)_4$ were added to the solution under stirring at room temperature for 15 h. The precipitate formed was separated by centrifugation, dried and calcined at 700, 800, 900 or 1000 °C for 4 h. The catalysts obtained were denoted as ZrO_2_27, ZrO_2_28, ZrO_2_29 or ZrO_2_30. ZrO_2_31 and ZrO_2_32 were prepared using zirconium(IV) propoxide solution (70 wt% in 1-propanol, ChemPur) as a precursor. Typically, 23.398 g of zirconium(IV) propoxide solution were mixed with 11.7 mL of ethanol and 5.2 mL of 25% aqueous ammonia solution. The resulting mixture was heated up to 85 °C and stirred for 10 min. Hereafter, 2.4 mL of H_2O were added under stirring for 50 min until gelation occurred. The sample was separated by filtering, initially dried at room temperature for 24 h and then at 100 °C for 24 h. Finally, the solid was calcined at 900 and 1000 ^{o}C for 5 h. The catalysts were denoted as ZrO_2_31 and ZrO_2_32 respectively.

ZrO_2_33 was prepared by filter paper templating approach according to Ref. [2]. To this end, 28.0271 g of $ZrO(NO_3)_2 \cdot xH_2O$ were dissolved in 81.2 mL of H_2O. The obtained solution was used to impregnate 38.667 g of filter paper 50 (Whatman, 1450 125) cut into small pieces about 3 × 1 cm). After that, the wet filter paper was directly calcined at 550 °C for 4 h. The obtained solid was calcined at 900 °C for 5 h.

ZrO_2_34 was prepared by calcination of $ZrO(NO_3)_2 \cdot xH_2O$ at 1000 °C for 5 h.

ZrO_2_35 was provided by Saint-Gobain without any treatment. ZrO_2_36, ZrO_2_37, ZrO_2_38 were prepared by calcination of ZrO_2_35 at 550, 700, and 900 °C for 4 h respectively.

Supplementary Note 2

Temperature-programmed desorption (TPD) of CO was carried out in an in-house developed setup containing eight individually heated continuous-flow fixed-bed quartz reactors. The following procedure was applied. The catalysts (0.05 g) were initially calcined in air at 550 °C for 1 h, purged with Ar for 15 min and then treated with 57 vol% H_2 in Ar flow for 1 h. Hereafter, the treated catalysts were cooled down in Ar flow to 250 °C and then exposed to a flow of CO (1 vol% CO in Ar, 10 $mL \cdot min^{-1}$) for 1 h followed by cooling down in Ar flow to room temperature. Finally, the catalysts were heated to 900 °C with a heating rate of 10 $K \cdot min^{-1}$ in Ar flow (10 $mL \cdot min^{-1}$). Desorbed CO was registered by an on-line mass spectrometer (Pfeiffer Vacuum OmniStar GSD 320) at the atomic mass unit (AMU) of 28.

To investigate acidic properties of ZrO_2, NH_3 or C_3H_6 were used as probe molecules for TPD tests. The catalysts (0.05 to 0.2 g) were initially calcined in air at 550 °C for 1 h, purged with Ar for 15 min and then treated with 57 vol% H_2 in Ar flow at the same temperature for 1 h. After that, the catalysts were cooled down in Ar flow to 120 (for NH_3 adsorption) or to 50 °C (for C_3H_6 adsorption). Hereafter, they were exposed to a flow of either 1 vol% NH_3 in Ar (14 $mL \cdot min^{-1}$) or 5 vol% C_3H_6 in Ar (6 $mL \cdot min^{-1}$) for 1 h. The catalysts for NH_3-TPD were then cooled down in Ar flow to 80 °C, tempered for 2 h and finally heated to 900 °C with a heating rate of 10 $K \cdot min^{-1}$. The catalysts for C_3H_6-TPD were purged in Ar flow at 50 °C for 12 h to remove weakly adsorbed C_3H_6 and finally heated to 900 °C with a heating rate of 10 $K \cdot min^{-1}$. Desorbed ammonia or propene was registered by an on-line mass spectrometer (Pfeiffer Vacuum OmniStar GSD 320) at AMU of 15 (NH) or AMU of 41 (C_3H_5) and 40(Ar).

Temperature-programmed reduction (TPR) experiments were performed using a feed with either 5 vol% H_2 or 1 vol% CO in Ar. Catalysts (0.2 g) were firstly calcined in air at 550 °C for 1 h, then cooled down in Ar flow to room temperature. Hereafter, they were heated in a flow of 5 vol% H_2 (10 mL·min^{-1}) or 1 vol% CO (10 mL·min^{-1}) in Ar from room temperature to 900 °C with a heating rate of 10 K·min^{-1}. The consumption of H_2 or CO was quantified with an on-line mass spectrometer (Pfeiffer Vacuum OmniStar GSD 320).

The electrical conductivity measurements of the catalysts were performed in a fixed-bed continuous-flow tubular quartz reactor. Firstly, each catalyst was pressed into a dense disc of 7.1 mm in diameter and 1.2-1.4 mm in height to minimize interior grain boundaries. Both surfaces of the disc were covered with a platinum conducting paste (ChemPur, 71% Pt), and dried at 120 °C. Hereafter, a sample disc was sandwiched between two inert platinum electrodes and arranged inside the reactor. A constant alternating current of 1 kHz was adjusted with an automatically compensating bridge (Wayne Kerr B905). At this mono-frequency regime, the contributions of both surface and bulk conductivity are regarded similar for all samples, owing to the identical treatment of the probed discs. As an equivalent circuit, a parallel connection of a condenser and an ohmic resistance was applied. Electrical conductivity of catalysts was measured at 550 °C in air and N_2 to ensure different partial pressures of oxygen, i.e. 20 kPa and about 10^{-4} kPa respectively. The electrical conductivity of the catalysts was calculated according to Supplementary Equation 1.

$$\sigma = \frac{1}{R} \times \frac{h}{S} \quad (1)$$

Where R is the electrical resistance (Ω), and the ratio h/S represents the thickness h (mm) and cross section area S (mm^2) of the catalyst disc.

Supplementary Note 3

Catalytic tests with C_2H_6, C_3H_8, and iso-C_4H_{10} were performed at 1 bar between 550 and 625°C using an in-house developed setup consisting of 15 continuous-flow fixed-bed quartz tubular (length and inner diameter are 465 and 4 mm respectively) reactors operating in parallel. Tests with CH_4 were carried out at 800°C and 1.25 bar in an in-house developed setup consisting of 6 continuous-flow fixed-bed quartz tubular (length and inner diameter are 330 and 4 mm respectively) reactors operating in parallel. To determine the rate of olefin formation, the tests were carried out at a degree of alkane conversion below 10% using a feed containing 40 vol% C_mH_{2m+2} (m=1, 2, 3, or 4) in N_2. The mass (0.05 to 0.3 g) of catalyst and total flow (10 to 40 mL·min^{-1}) were varied to fulfil this requirement. The catalysts were firstly heated in N_2 flow up to 550 °C (in case of C_2H_6, C_3H_8 and iso-C_4H_{10}) or to 800°C (in case of CH_4) followed by calcination in air flow for 1 h. Then they were purged with N_2 for 15 min, treated with H_2 or CO flow (57 vol% H_2 or CO in N_2) for 1 h and flushed in N_2 for 15 min. Finally, a flow of C_mH_{2m+2}-N_2 mixture (40 vol% C_mH_{2m+2} in N_2) was passed through the catalyst bed. To investigate the influence of the nature of reducing agent (H_2 or CO) and the effect of reduction time on catalyst activity, the reduction time varied from 0 to 420 min and from 0 to 50 min for H_2 and CO respectively. The initial rate of olefin formation in tests with CH_4, C_2H_6, C_3H_8 or iso-C_4H_{10} was measured after 600, 600, 190 or 540 s on stream.

To determine an integral propene selectivity (Supplementary Equation 2), catalytic tests were performed at 550 °C for 1 hour. Total flow of a mixture of 40 vol% C_3H_8 in N_2 was fixed at 10 mL·min^{-1}, catalyst amount (0.12 to 2.4 g) was varied to achieve an initial propane conversion of 30%. On stream profiles of propene yield and propane conversion in Supplementary Figure 4

were integrated to obtain the number of moles of propene formed ($n(C_3H_6)_{formed}$) and the number of moles of propane consumed ($n(C_3H_8)_{consumed}$) within 1 hour propane dehydrogenation.

$$S(C_3H_6) = \frac{n(C_3H_6)_{formed}}{n(C_3H_8)_{consumed}} \tag{2}$$

Long-term stability of ZrO_2 with 9.1 nm crystallites (ZrO_2_35 in Supplementary Table 1) was checked in a series of 70 PDH/regeneration cycles. The cycles were performed at 550, 600 or 625 °C using total flow of C_3H_8-N_2 mixture of 10, 40 or 60 $mL \cdot min^{-1}$. The catalyst amount was set to 0.3 g. Prior to the first PDH stage, the catalyst was heated in a flow of air at 550 °C for 1 h. The PDH stage lasted for 38 min followed by flushing in N_2 flow for 15 min and then treating in air flow for 15 min.

To determine the dependence of product selectivity on propane conversion, we performed catalytic tests at 550 °C with the catalysts reduced in a flow of H_2 (57 vol% H_2 in N_2). Catalyst amount (0.25 to 2.1 g) and total flow (6 to 80 $mL \cdot min^{-1}$) were varied to get different contact time and accordingly different degree of propane conversion.

The feed components and the reaction products were analysed by an on-line gas chromatograph (Agilent 6890) equipped with PLOT/Q (for CO_2), AL/S (for hydrocarbons), and Molsieve 5 (for H_2, O_2, N_2, and CO) columns as well as flame ionization and thermal conductivity detectors.

Supplementary Equations 3-8 were used to calculate the rate of olefin formation ($r(C_nH_{2n})$ expressed as $mmol(C_nH_{2n}) \cdot g^{-1}_{cat} \cdot min^{-1}$), alkane conversion ($X(C_mH_{2m+2})$), selectivity to gas-phase products (S_i) and coke (S(coke)), olefin yield ($Y(C_nH_{2n})$), and space time yield of olefin

formation ($STY(C_nH_{2n})$ expressed as $kg(C_nH_{2n})\cdot h^{-1}\cdot kg^{-1}$) respectively. n is 2, 3 or 4, while m is 1, 2, 3, or 4.

$$r\left(C_nH_{2n}\right)=\frac{STY\left(C_nH_{2n}\right)\cdot 1000}{60\cdot M\left(C_nH_{2n}\right)} \tag{3}$$

$$X\left(C_mH_{2m+2}\right)=\frac{\dot{n}^{in}_{C_mH_{2m+2}}-\dot{n}^{out}_{C_mH_{2m+2}}\cdot\frac{\dot{n}^{in}_{N_2}}{\dot{n}^{out}_{N_2}}}{\dot{n}^{in}_{C_mH_{2m+2}}} \tag{4}$$

$$S_i=\frac{v_{C_mH_{2m+2}}}{v_i}\cdot\frac{\dot{n}^{out}_i\cdot\frac{\dot{n}^{in}_{N_2}}{\dot{n}^{out}_{N_2}}}{\dot{n}^{in}_{C_mH_{2m+2}}-\dot{n}^{out}_{C_mH_{2m+2}}\cdot\frac{\dot{n}^{in}_{N_2}}{\dot{n}^{out}_{N_2}}} \tag{5}$$

$$S\left(coke\right)=1-\sum S_i \tag{6}$$

$$Y\left(C_nH_{2n}\right)=X\left(C_mH_{2m+2}\right)\cdot S\left(C_nH_{2n}\right) \tag{7}$$

$$STY\left(C_nH_{2n}\right)=\frac{F_{feed}\cdot x\left(C_nH_{2n}\right)\cdot M\left(C_nH_{2n}\right)}{V_m\cdot m_{cat}} \tag{8}$$

Here, F_{feed} is a volumetric feed flow rate ($mL\cdot h^{-1}$), $x(C_nH_{2n})$ is a molar fraction of olefin, V_m is molar volume (22400 $mL\cdot mol^{-1}$), m_{cat} is catalyst amount (g), $\dot{n}$ with superscripts "in" and "out" is a molar flow of gas phase components (indicated with subscripts "i", "C_mH_{2m+2}" or "N_2") at the reactor inlet and outlet respectively ($mol\cdot min^{-1}$), v_i is reciprocal stoichiometric coefficient for product i, $M(C_nH_{2n})$ is a molar weight of C_nH_{2n} ($g\cdot mol^{-1}$).

Supplementary Methods

Spin-polarized and periodic density functional theory (DFT) calculations were carried out by using the Vienna *ab initio* simulation package (VASP)[3, 4]. Exchange and correlation were treated within the Perdew-Burke-Ernzerhof generalized gradient approximation (GGA-PBE)[5]. To obtain accurate energies with errors of less than 1 meV per atom, a cutoff energy of 400 eV was used. Geometry optimization was converged until forces acting on atoms were lower than 0.02 eV/Å, whereas the energy threshold defining self-consistency of the electron density was set to 10^{-4} eV. The Climbing Image Nudged Elastic Band (CI-NEB) method with eight images was applied for finding transition states and minimum energy paths of all reactions[6]. The final transition state structures were refined by using the quasi-Newton algorithm until the Hellman-Feynman forces on each ion were lower than 0.02 eV/Å. The normal mode frequency analysis was performed to validate the optimized transition states and each authentic transition state has only one imaginary frequency along the reaction coordinates. The resulted zero-point vibrational energies (ZPE) from the frequency analysis are included in our energetic comparison and discussion. For the optimization of the bulk structure, the lattice parameters of the monoclinic ZrO_2 (m-ZrO_2) phase were determined by minimizing the total energy of the unit cell by using a conjugated gradient algorithm to relax the ions. A 7×7×7 Monkhorst−Pack k point grid was used for sampling the Brillouin zone[7]. In addition, we also tested the corrections of Hubbard term (PBE+*U*) and dispersion (DFT+D3). These new data are now presented in Supplementary Figure 22 and Supplementary Figure 23.

Previous works[8, 9] pointed out that generalized gradient approximation (GGA) functional (such as PBE) underestimate the energy of the occupied Zr 4d orbital, and correspondingly the band gap of the bulk material. To account for this effect, a Hubbard *U*-like term describing the

on-site Coulomb interactions of 4 eV is required[9]. We applied PBE+U to the Zr 4d orbitals, with U_{eff} values ranging from 0-6 eV according to the approach defined by Dudarev et al.[10] In particular, U_{eff} is defined as the difference between the Coulomb energy, U, and the exchange parameter, J. PBE+U calculations were performed for the oxygen vacancy formation energy (E_{vac}) and the C_3H_8 dissociation on Zr_{6c}–O_{2c} site of $ZrO_2(\bar{1}11)$ to form isopropyl and surface hydroxyls (E_{ads}), a key intermediate in the studied reaction (Supplementary Figure 22). The energy difference between the values derived from PBE or PBE+U calculations, both for E_{vac} and E_{ads}, is always smaller than 0.18 eV, for any U_{eff} value. Due to the negligible influence of the U_{eff} term on the magnitudes of relevance for our work, the PBE functional has been applied with no on-site Hubbard term.

Dispersion interactions are modelled using the DFT-D3 method developed by Grimme et al[11]. Previous works concluded that this method can provide accurate estimates of the adsorption energies of n-alkanes on PdO(101) and $RuO_2(110)$ in comparison with TPD derived values[12, 13]; while the DFT-D3 calculations overestimate the adsorption energy of CH_4 and C_2H_6 on the $IrO_2(110)$ surface[14, 15]. We established that DFT-D3 calculations using the PBE functional may overestimate the binding energies of C_3H_8 and C_3H_6 on s-$ZrO_2(\bar{1}11)$. The results of DFT-PBE calculations performed with dispersion corrections in Supplementary Figure 23 indicate that the predictions from both methods support the conclusions of this study.

As XRD analysis proved that our catalysts mainly contain the monoclinic phase, we also calculated monoclinic ZrO_2 (m-ZrO_2) models. The calculated lattice parameters of the unit cell are a = 5.144 Å, b = 5.262 Å, c = 5.285 Å and β = 99.36° and are in excellent agreement with the experimental values (a = 5.151 Å, b = 5.212 Å, c = 5.317 Å and β = 99.23°)[16] as well as with

other theoretical results[17-21] in Supplementary Table 6 thus validating our computational parameters.

Among all m-ZrO_2 surfaces, the $(\bar{1}11)$ termination with O-Zr-O surface is most stable[22, 23]. The m-$ZrO_2(\bar{1}11)$ surface was modelled by a nine-layer slab with the lowest three layers fixed in the bulk positions and the upper six layers being allowed to relax. In all cases, a $p(2\times2)$ lateral supercell containing a vacuum space of 15 Å between the slab and its periodic replicas was used. The stoichiometric m-$ZrO_2(\bar{1}11)$ surface exhibits seven- and six-fold coordinated Zr atoms (Zr_{7c} and Zr_{6c}) as well as two- and three-fold coordinated oxygen atoms (O_{2c} and O_{3c}). In order to assist the description (Supplementary Figure 16), hereinafter, we denoted the four Zr centres as Zr_{6c}^{I}, Zr_{7c}^{II}, Zr_{6c}^{III}, and Zr_{6c}^{IV} and the four O_{3c} cations as O_{3c}^{I}, O_{3c}^{II}, O_{3c}^{III}, and O_{3c}^{IV} respectively. The Zr–O_{2c} site for initial C_3H_8 dissociation is denoted as Zr_{6c}^{I}–O_{2c} (Supplementary Figure 16). The Brillouin zone was sampled with a 2×2×1 Monkhorst–Pack mesh.

The oxygen-imperfect m-$ZrO_2(\bar{1}11)$ surface was also computed. Surface oxygen vacancy (O_v) was created by removal of a neutral oxygen atom. The vacancy formation energy, $E_f(O_v)$, was calculated according to equation $E_f(O_v) = E_d + 1/2E_{O2} – E_s$, where E_d is the energy of oxygen defect surface slab and E_S is the energy of the clean surface slab, while E_{O2} is the energy of an isolated gaseous O_2 molecule. The oxygen vacancy formation energy is a measure of the reducibility of the material. The higher the (positive) value, the lower the reducibility.

The oxygen vacancy formation energies for $(\bar{1}11)$ surface corresponding to a surface concentration of one vacancy per five possible oxygen sites (Supplementary Table 3) vary between 5.70 and 6.08 eV. Removal of O_{2c} was established to be the most preferential way to

create an oxygen vacancy. In the following, we investigated propane dehydrogenation on defective *m*-$ZrO_2(\bar{1}11)$ surface with the O_{2c} removed.

In addition, we tested the possibility of using H_2 and CO as a reduction agent to create oxygen vacancy according to ΔE_r (H_2/CO) = E_d + $E(H_2O/CO_2)$ – E_s + $E(H_2/CO)$. Thermodynamically, it is found that oxygen vacancy formation using CO is more favored than using H_2, indicated by 0.73 eV energy difference. This is indeed in agreement with our experiment, where it is found that catalyst reduced in CO shows much higher activity than that reduced in H_2, indicating the easier formation of oxygen vacancy as well as the higher concentration of oxygen vacancy by using CO instead of H_2.

The adsorption energy is defined by $E_{ads} = E_{X+s/d} – E_{s/d} – E_X$, where $E_{X+s/d}$ is the total energy of the slab (clean and oxygen defect surface) with adsorbates in its equilibrium geometry, and E_X is an isolated gaseous adsorbate molecule. The more negative the adsorption energy, the stronger the adsorption. The activation barrier (or activation energy, E_a) and ΔE_r are calculated according to $E_a = E_{TS} - E_{IS}$ and $\Delta E_r = E_{FS} - E_{IS}$, where E_{IS}, E_{FS} and E_{TS} are the energies of the corresponding initial state (IS), final state (FS) and transition state (TS) respectively.

Methylene C–H activation over s-$ZrO_2(\bar{1}11)$ (Supplementary Table 7) can occur either heterolytically or homolytically. Heterolytic C–H activation involves the Zr–O sites [Zr_{6c}^{I}–O_{2c}, O_{2c}–Zr_{6c}^{I}, Zr_{6c}^{I}–O_{3c}^{I}, Zr_{6c}^{I}–O_{3c}^{II}. Zr_{6c}^{I}–O_{3c}^{III}, Zr_{6c}^{III}–O_{3c}^{II}, Zr_{6c}^{III}–O_{3c}^{III}, Zr_{6c}^{IV}–O_{3c}^{I}, Zr_{6c}^{IV}–O_{3c}^{II} and Zr_{6c}^{IV}–O_{3c}^{III}] and results in Zr–$C^{\delta-}$ and O–$H^{\delta+}$ intermediates [C_3H_8 + Zr–O → Zr–*iso*-C_3H_7 + O–H]. Homolytic C–H activation involves a basic lattice oxygen atom as the active site, which abstracts a hydrogen atom and results in a weakly coordinated isopropyl species which then rebinds with a neighbouring oxygen. The reaction occurs over two surface oxygen atoms (O_{2c}–

O_{3c}^{I} and O_{3c}^{I}–O_{2c} sites) forming O–*iso*-C_3H_7 and O–H. The methylene C–H dissociative adsorption energies suggest that the Zr_{6c}^{I}–O_{2c} site is least endothermic (0.94 eV). We also compared the methyl C–H activation at the Zr_{6c}^{I}–O_{2c} site and this step is endothermic by 0.86 eV.

On the basis of the computed C–H dissociative adsorption energy (Supplementary Table 7) we considered the dehydrogenation of propane to propene firstly via methylene C–H activation (Fig. 6 in the main manuscript and Supplementary Figure 18). This heterolytic step has a barrier of 1.25 eV and is endothermic by 0.94 eV at the Zr_{6c}^{I}–O_{2c} site. In the transition state (TS1), the dissociating H atom is located between the O_{2c} and C atoms, and the length of the C–H and O_{2c}–H bonds is 1.559 and 1.132 Å respectively. The formed C–Zr_{6c}^{I} bond has a length of 2.468 Å. In the dissociated state (C2), the isopropyl group is located at the Zr_{6c}^{I} centre with the C–Zr_{6c}^{I} distance of 2.330 Å and the H atom is at the adjacent O atom in form of OH group.

Subsequently, breakage of one methyl C–H bond of the isopropyl group occurs at Zr_{6c}^{III} with the hydrogen atom between the adjacent Zr_{6c}^{III} and Zr_{6c}^{I} atoms (TS2). The length of the C–H bond is 1.725 Å and the length of the formed Zr_{6c}^{III}–H and Zr_{6c}^{I}–H bonds is 2.134 and 2.163 Å respectively. This reaction step has a barrier of 0.74 eV and is endothermic by 0.46 eV.

Since propene has very low adsorption energy (0.13 eV) and can desorb easily from the surface immediately after its formation (D2), we therefore considered H_2 formation directly. After propene desorption (E2), one H atom is located at O_{2c} (O_{2c}–H) and another H atom is located between Zr_{6c}^{III} and Zr_{6c}^{I} (Zr_{6c}^{III}–H = 2.006 Å and Zr_{6c}^{I}–H = 2.484 Å). The recombination of two H atoms requires the diffusion of the H atom linking Zr^{III} and Zr^{I}. In the transition state (TS3), the distance of Zr_{6c}^{III}–H and Zr_{6c}^{I}–H becomes 3.031 and 1.930 Å respectively. This step has a barrier of 0.38 eV and is slightly endothermic by 0.27 eV. The final H_2 formation has a

barrier of 0.20 eV and is exothermic by 0.55 eV. In the transition state (TS4), the forming H–H bond has a length of 1.049 Å; and the length of the breaking Zr_{6c}^{I}–H and O_{2c}–H bond is 2.050 and 1.208 Å respectively.

For comparison, we computed the methyl C–H dissociation as the first step and subsequently the central methylene C–H dissociation as the second step. For the first step, the reaction has a barrier of 1.23 eV and is endothermic by 0.86 eV; and these values are very close to those for methylene C–H dissociation as the first step. For the second step, however, it is not possible to locate the corresponding transition state of the central methylene C–H dissociation due to its upright adsorption configuration (C2'), from which it needs to bend the C–C–Zr angle (119.9°) very strongly and thus much high barrier. It is also to note that there are six C–H bonds in isopropyl group to dissociate, while there are only two C–H bond to dissociate in *n*-propyl group.

Methylene C–H activation over d-$ZrO_2(\bar{1}11)$ (Supplementary Table 8) also occurs heterolytically via the Zr–O site and homolytically via the (Zr, Zr)–O_v site. Heterolytic C–H dissociation at the Zr–O sites (Zr_{5c}^{I}–O_{3c}^{I}, Zr_{5c}^{I}–O_{3c}^{II}, Zr_{5c}^{I}–O_{3c}^{III}, Zr_{6c}^{III}–O_{3c}^{II}, Zr_{6c}^{III}–O_{3c}^{III}, Zr_{6c}^{IV}–O_{3c}^{I}, Zr_{6c}^{IV}–O_{3c}^{II} and Zr_{6c}^{IV}–O_{3c}^{III}) is endothermic, similar to that on the s-ZrO_2 surface. While homolytic methylene C−H activation on the [Zr_{5c}^{I}, Zr_{6c}^{II}]–O_v and [Zr_{5c}^{I}, Zr_{6c}^{III}]–O_v sites of d-ZrO_2 is strongly exothermic by 1.04 and 0.55 eV respectively, and the ([Zr_{5c}^{I}, Zr_{6c}^{II}]–O_v site is thermodynamically more favoured than the [Zr_{5c}^{I}, Zr_{6c}^{III}]–O_v site.

Since homolytic methylene C−H activation on the [Zr_{5c}^{I}, Zr_{6c}^{II}]–O_v and [Zr_{5c}^{I}, Zr_{6c}^{III}]–O_v sites of d-ZrO_2 is strongly exothermic (1.04 and 0.55 eV respectively), and the [Zr_{5c}^{I}, Zr_{6c}^{II}]–O_v site is thermodynamically more favoured than the [Zr_{5c}^{I}, Zr_{6c}^{III}]–O_v site, we computed the

dissociation energy barrier of the two reaction pathways (Supplementary Figure 19). It is found that the [Zr_{5c}^{I}, Zr_{6c}^{II}]–O_v site (black) has a lower barrier than the [Zr_{5c}^{I}, Zr_{6c}^{III}]–O_v site (orange) (0.16 vs. 0.73 eV). The length of the C−H bond of TS1 activated at the [Zr_{5c}^{I}, Zr_{6c}^{II}]–O_v and [Zr_{5c}^{I}, Zr_{6c}^{III}]–O_v sites is 1.428 and 1.515 Å respectively (1.103Å of gaseous C_3H_8). After the dissociation, the iso-propyl group is at the Zr_{5c}^{I} site and the H atom is bridging Zr_{5c}^{I} and Zr_{6c}^{II} (Zr_{5c}^{I}–H–Zr_{6c}^{II}).

After methylene C–H dissociation (C1), we computed the subsequent methyl C–H dissociation (Supplementary Figure 21). It is found that methyl C–H dissociation by adjacent Zr_{6c}^{III} has a barrier of 0.67 eV and is endothermic by 0.42 eV with the hydrogen atom between the adjacent Zr_{6c}^{III} and Zr_{5c}^{I} atoms (TS2). The length of the breaking C–H bond is 1.708 Å and the length of the forming Zr_{6c}^{III}–H and Zr_{5c}^{I}–H bonds is 2.242 and 2.129 Å respectively. Since propene has low adsorption energy (0.46 eV) on this hydrogen adsorbed surface and can desorb easily from the surface immediately after its formation (D1), we considered H_2 formation directly. After propene desorption (E1), one H atom is located between Zr_{6c}^{II} and Zr_{5c}^{I} (Zr_{5c}^{II}–H = 2.075 Å and Zr_{5c}^{I}–H = 2.104 Å) and another H atom is located between Zr_{6c}^{III} and Zr_{5c}^{I} (Zr_{6c}^{III}–H = 1.997 Å and Zr_{5c}^{I}–H = 2.542 Å). The recombination of two H atoms requires the diffusion of the H atom linking Zr_{6c}^{III} and Zr_{5c}^{I}. In the transition state (TS3), the distance of Zr_{6c}^{III}–H and Zr_{5c}^{I}–H becomes 2.990 and 1.934 Å respectively. This step has a barrier of 0.28 eV. The final H_2 formation is endothermic by 1.40 eV (H_2 dissociation is barrier-less) (G1).

In addition, we calculated the Gibbs energy G at the reaction temperature of experimental PDH tests, i.e. at 823.15 K and ambient pressure according to Supplementary Equation 9.

$$G(\mathrm{T, P}) = E_{\mathrm{e}} + E_{\mathrm{vib}} + E_{\mathrm{rot}} + E_{\mathrm{trans}} + PV - T(S_{\mathrm{vib}} + S_{\mathrm{rot}} + S_{\mathrm{trans}}) \quad (9)$$

The vibrational, rotational and translational contributions to enthalpy and entropy for a gas phase molecule considered as an ideal gas can be calculated using Supplementary Equations 10-16.

$$E_{\mathrm{vib}} = R\sum_{n} \frac{h\nu_{n}}{k_{B}}\left(\frac{1}{2} + \frac{1}{\exp\left(\frac{h\nu_{n}}{k_{B}T}\right) - 1}\right) \tag{10}$$

$E_{\mathrm{rot}} + E_{\mathrm{trans}} + PV = 7/2RT$ (For linear molecules) (11)

$E_{\mathrm{rot}} + E_{\mathrm{trans}} + PV = 4RT$ (For non-linear molecules) (12)

$$S_{\mathrm{vib}} = R\sum_{n}\left[\frac{h\nu_{n}/k_{B}T}{\exp\left(\frac{h\nu_{n}}{k_{B}T}\right) - 1} - \ln(1 - \exp\left(\frac{-h\nu_{n}}{k_{B}T}\right)\right] \tag{13}$$

$S_{\mathrm{rot}} = R(\ln q_{\mathrm{rot}}+1)$ (For linear molecule) (14)

where $$q_{\mathrm{rot}} = \frac{1}{\sigma}\left(\frac{8\pi^{2}k_{B}T}{h^{2}}\right)\times I$$

$S_{\mathrm{rot}} = R(\ln q_{\mathrm{rot}}+3/2)$ (For non-linear molecule) (15)

where $$q_{\mathrm{rot}} = \frac{\sqrt{\pi}}{\sigma}\left(\frac{8\pi^{2}k_{B}T}{h^{2}}\right)^{3/2} \times \sqrt{I_{\mathrm{x}} \times I_{\mathrm{y}} \times I_{\mathrm{z}}}$$

$$S_{\mathrm{trans}} = R\left(\ln q_{\mathrm{trans}} + \frac{5}{2}\right) \tag{16}$$

where $$q_{\mathrm{trans}} = \left(\frac{2\pi m k_{B}T}{h^{2}}\right)^{3/2}\frac{k_{B}T}{P}$$

where *I* is the moment of inertia, *s* is the rotational symmetry number and *m* is the mass of the molecule.

The translational, rotational, and vibrational and entropic contributions of gas-phase molecules were calculated on the basis of ideal gas models. However, it should be noted that

both propane and propene do not follow the ideal gas rule and this makes such a pressure and temperature dependent reaction more complicated than expected on the basis of the ideal gas models. For the adsorbed molecules and transition states on the surface, the rotational and translational contributions were converted into vibrational modes. We also approximated that the *PV* term is negligible because it is very small with regard to the energetic terms. Hence, the Gibbs energy for the surface species is computed from the simplified Supplementary Equation 17.

$$G(\mathrm{T, P}) = E_e + E_{vib} - T \times S_{vib} \tag{17}$$

Based on the established dominant reaction pathways of propane dehydrogenation to propene (Fig. 6 in the main manuscript), we calculated the Gibbs free energies and total energies of all relevant states (including transition states) along the reaction coordinate. The results obtained are shown in Supplementary Figure 17.

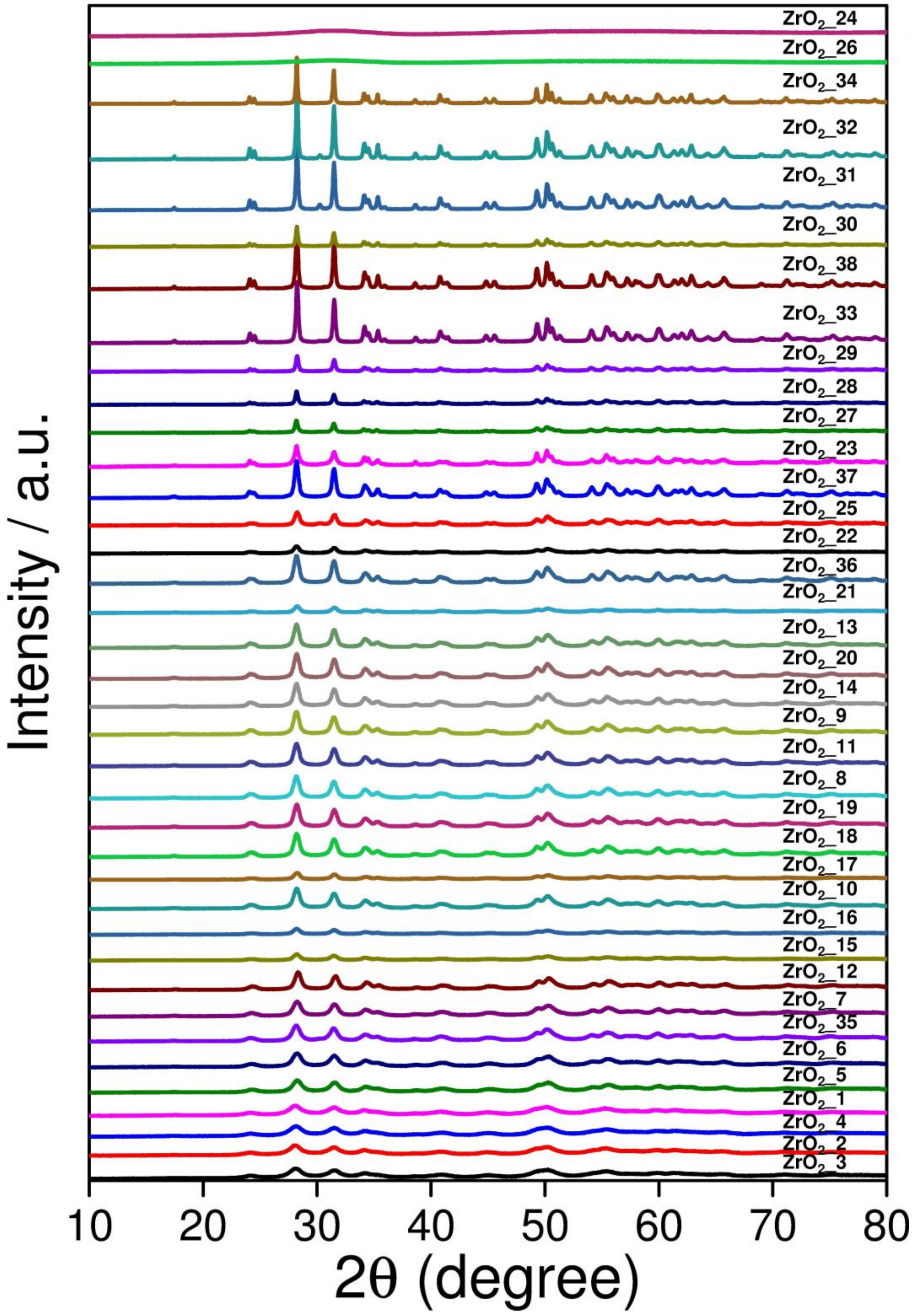

Supplementary Figure 1 X-Ray diffraction patterns of all ZrO_2 catalysts prepared by different methods (Supplementary Table 1). The first two top materials are amorphous. For other materials, the size of crystallites decreases from top to bottom.

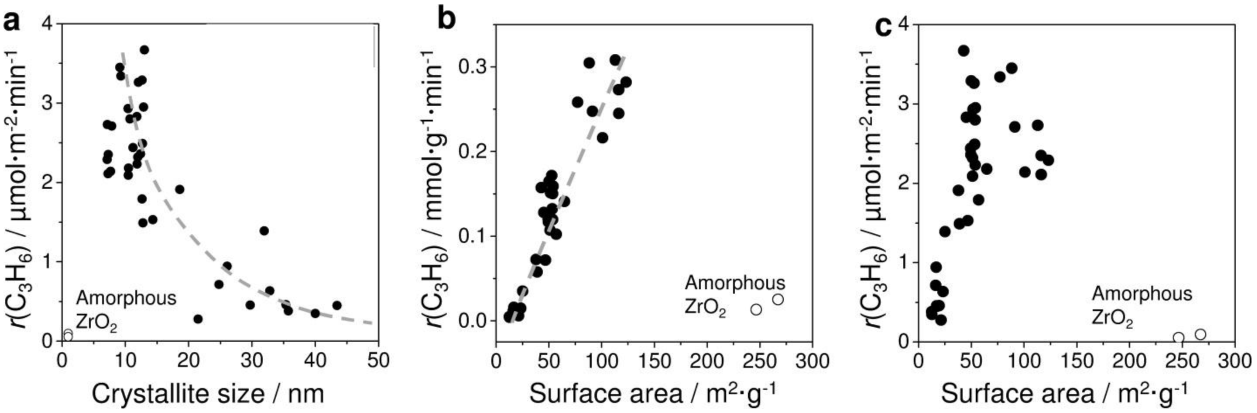

Supplementary Figure 2 The rate of propene formation related to **a** catalyst surface area versus the size of crystallites of differently synthesized ZrO_2 (Supplementary Table 1), **b** catalyst amount versus catalyst specific surface area and **c** catalyst surface area versus catalyst specific surface area. Reaction conditions: T=550 °C, feed containing 40 vol% C_3H_8 in N_2.

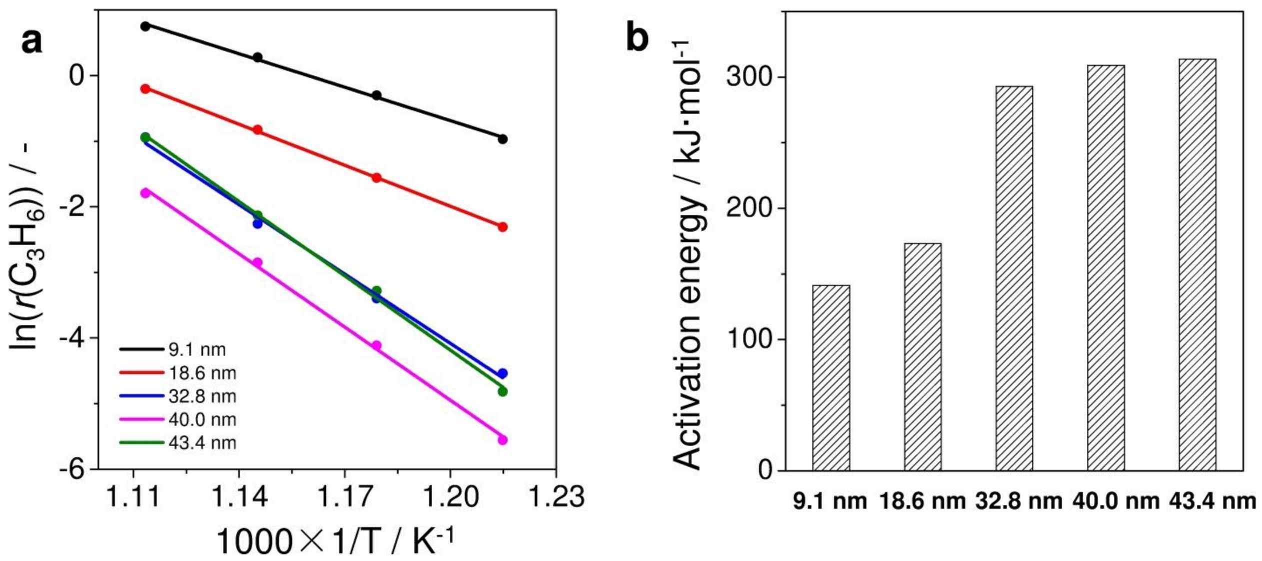

Supplementary Figure 3 a Arrhenius plots and **b** activation energy of propene formation over ZrO_2 with different size of crystallites. Reaction conditions: T=525-625 °C, feed containing 40 vol% C_3H_8 in N_2.

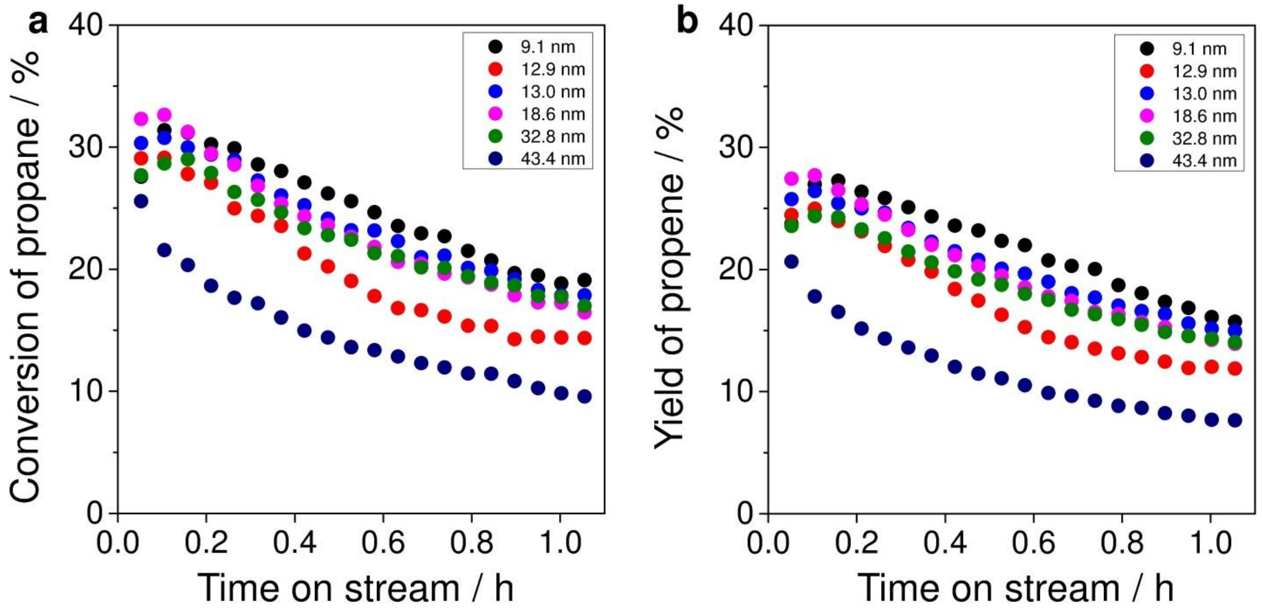

Supplementary Figure 4 Time on-stream profiles of **a** propane conversion and **b** propene yield obtained over ZrO_2 with different size of crystallites. Reaction conditions: T=550 °C, feed containing 40 vol% C_3H_8 in N_2.

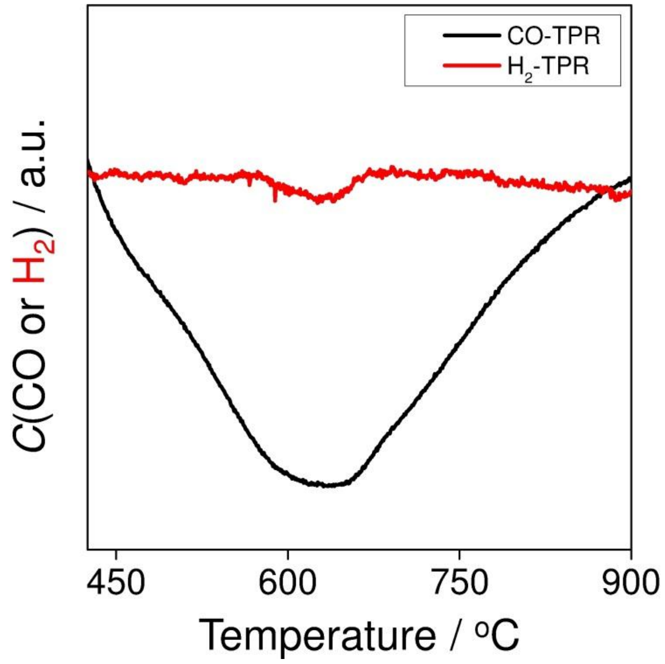

Supplementary Figure 5 CO-TPR (black line) and H_2-TPR (red line) profiles of ZrO_2 with 9.1 nm crystallites (ZrO_2_35 in Supplementary Table 1).

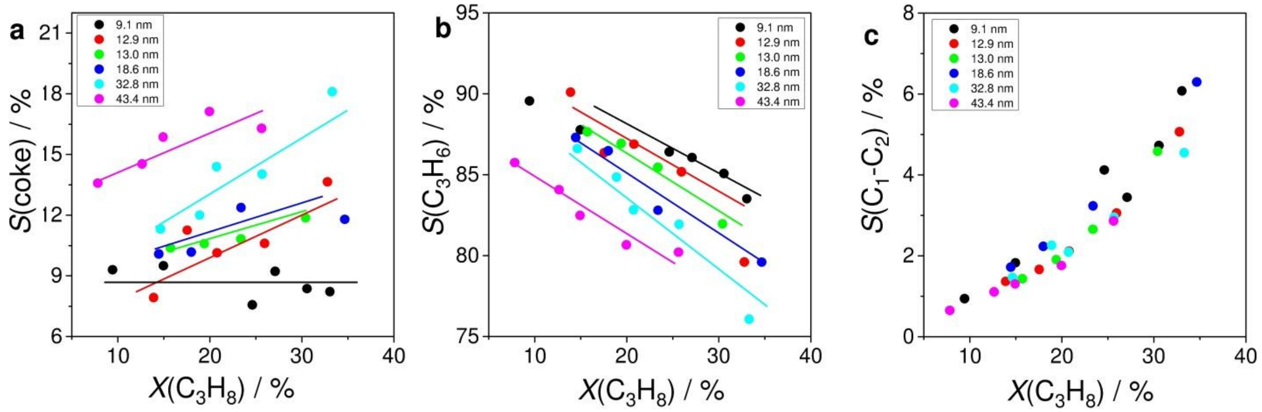

Supplementary Figure 6 Dependence of selectivity to **a** coke (S(coke)), **b** propene ($S(C_3H_6)$) and **c** cracking products ($S(C_1\text{-}C_2)$) on propane conversion over ZrO_2 with different crystallite size. Reaction conditions: T=550 °C, feed containing 40 vol% C_3H_8 in N_2.

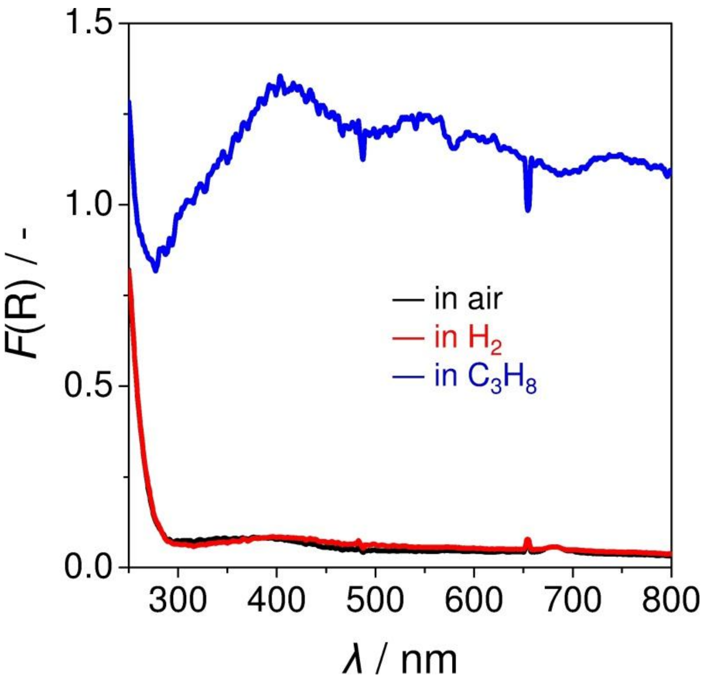

Supplementary Figure 7 UV-vis spectra of ZrO_2 at 550°C in air, after 60 min on H_2 stream (57 vol% H_2 in N_2) and after 60 min on C_3H_8 stream (40 vol% C_3H_8 in N_2).

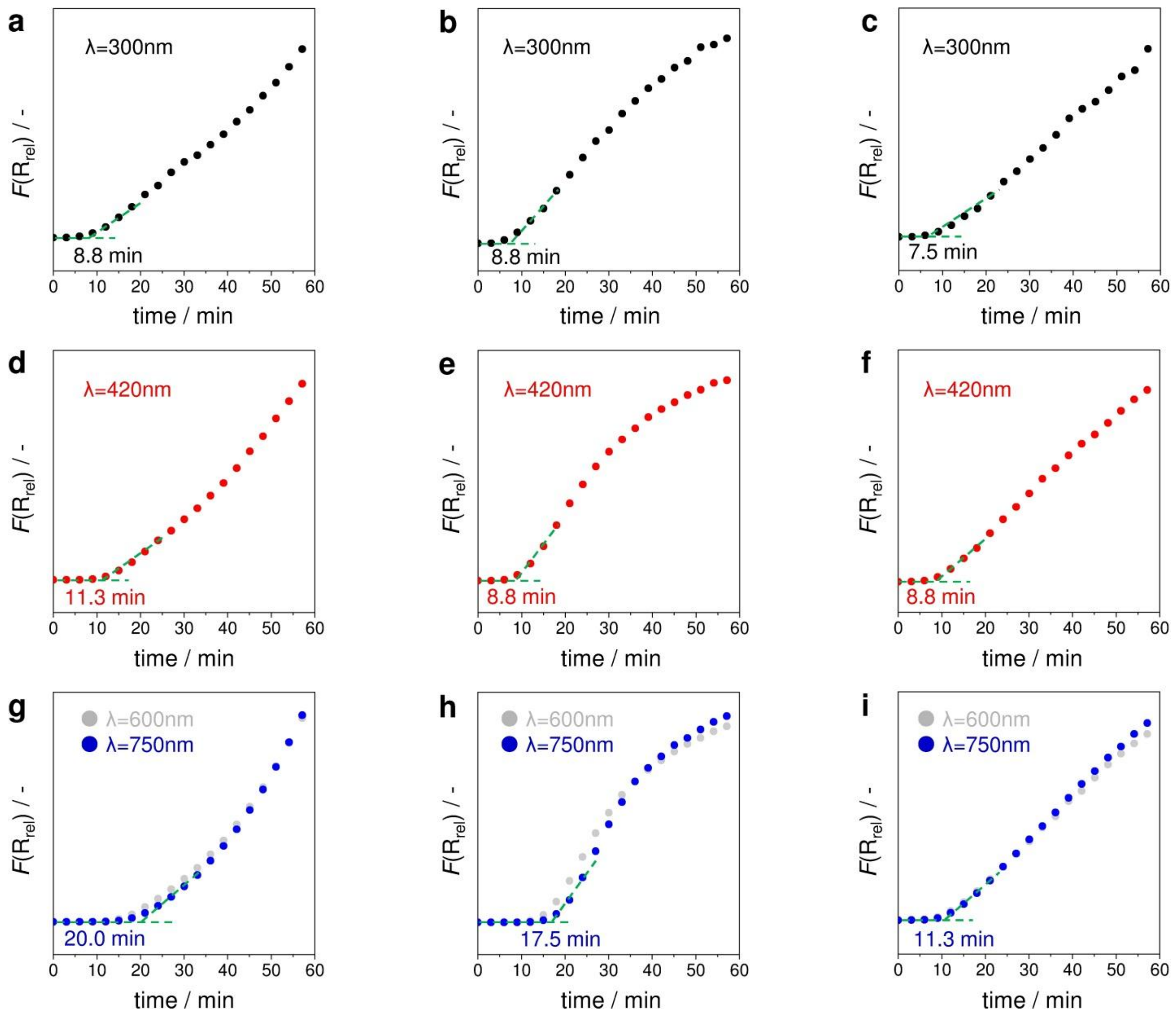

Supplementary Figure 8 Temporal changes in the Kubelka-Munk (F(R)) function at **a-c** 300, **d-f** 420, **g-i** 600 and **g-i** 750 nm upon catalyst treatment at 550°C in C_3H_8 (40 vol% C_3H_8 in N_2). ZrO_2 with crystallite size of **a, d, g** 9.1, **b, e, h** 13.0 and **c, f, i** 43.4 nm were investigated.

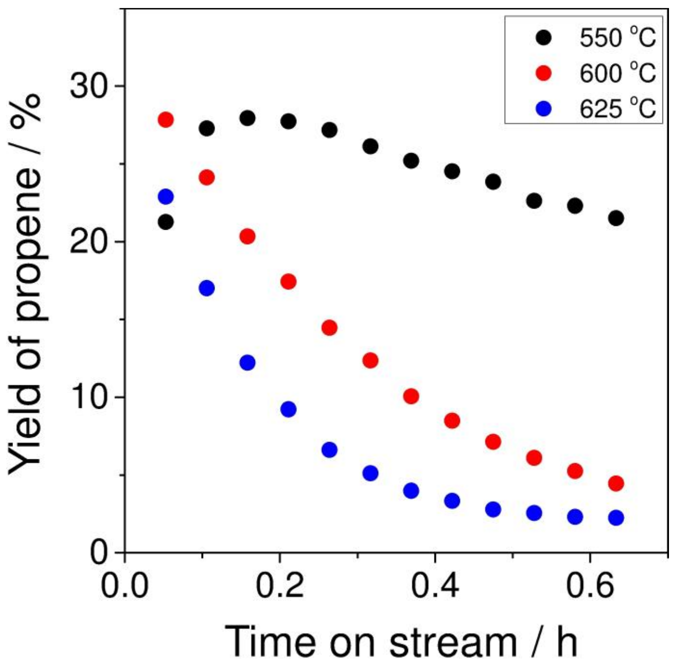

Supplementary Figure 9 Yield of propene versus time on stream over ZrO_2 with 9.1 nm crystallites (ZrO_2_35 in Supplementary Table 1) at 550 (black circle), 600 (red circle), and 625 °C (blue circle) using a reaction feed (40 vol% C_3H_8 in N_2) with the total flow of 10, 40 and 60 mL·min^{-1} respectively. Catalyst amount is 0.3 g.

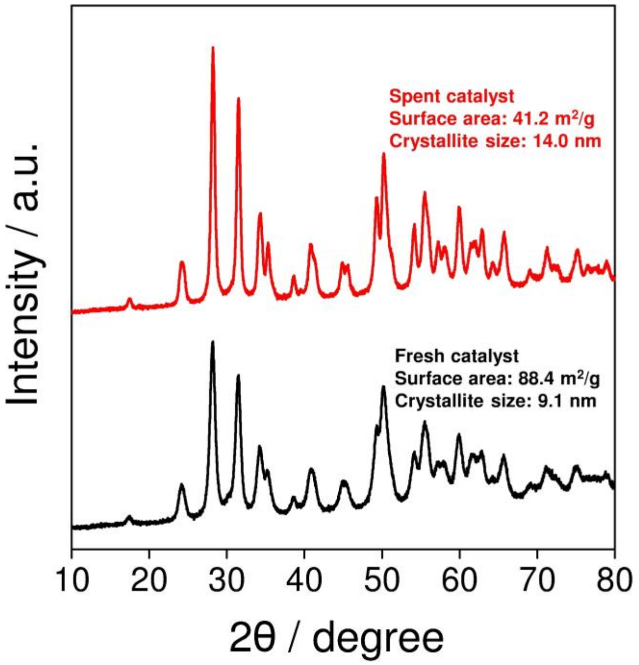

Supplementary Figure 10 X-Ray diffraction patterns of ZrO_2 (ZrO_2_35 in Supplementary Table 1). Black and red lines stand for the fresh and spent (after 70 PDH/regeneration cycles as defined in Fig. 4**b** in the main manuscript) catalysts.

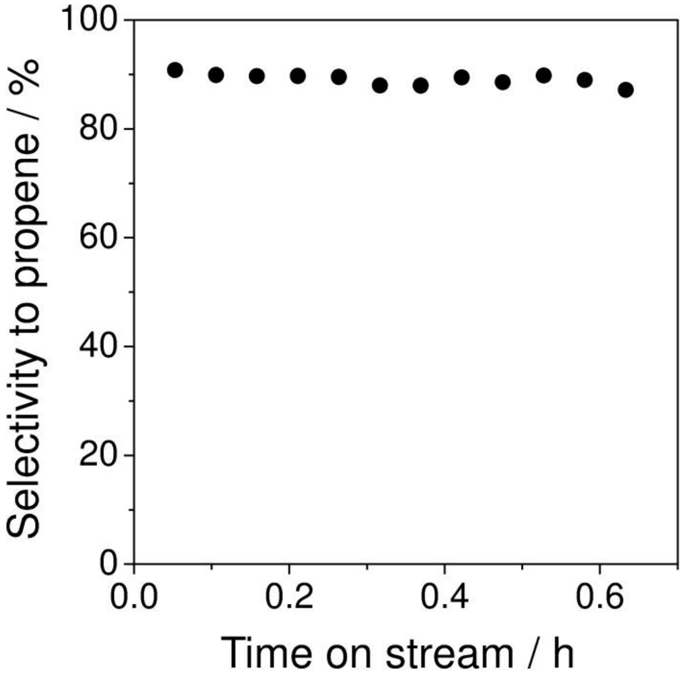

Supplementary Figure 11 Selectivity to propene versus time on stream over ZrO_2 with 9.1 nm crystallites (ZrO_2_35 in Supplementary Table 1) in the last PDH cycle of 70 PDH/regeneration cycles at 550 °C. For detail see Fig. 4**b** in the main manuscript.

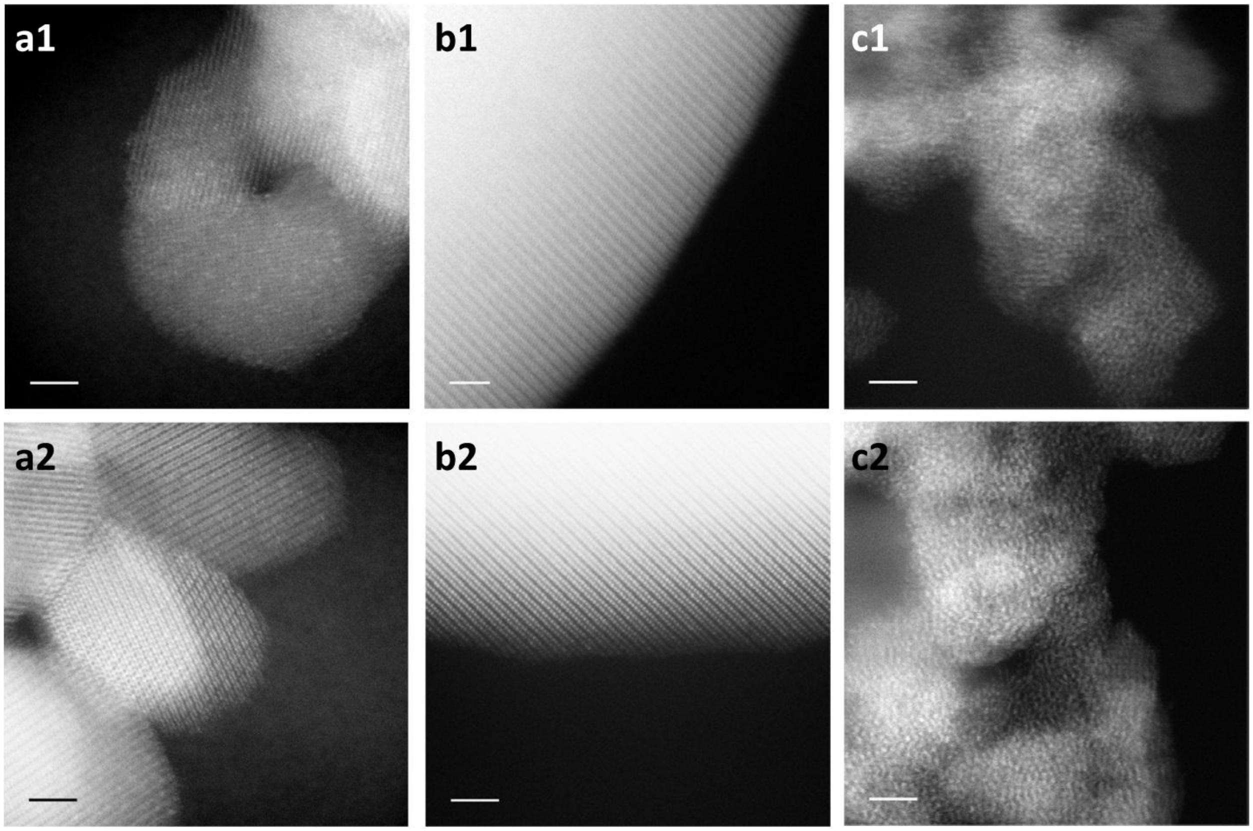

Supplementary Figure 12 High-resolution high-angle annular dark-field images of ZrO_2 with crystallites of **a1, a2** 9.1 nm and **b1, b2** 43.4 nm as determined by XRD as well as of **c1, c2** amorphous ZrO_2. Scale bars: 2 nm.

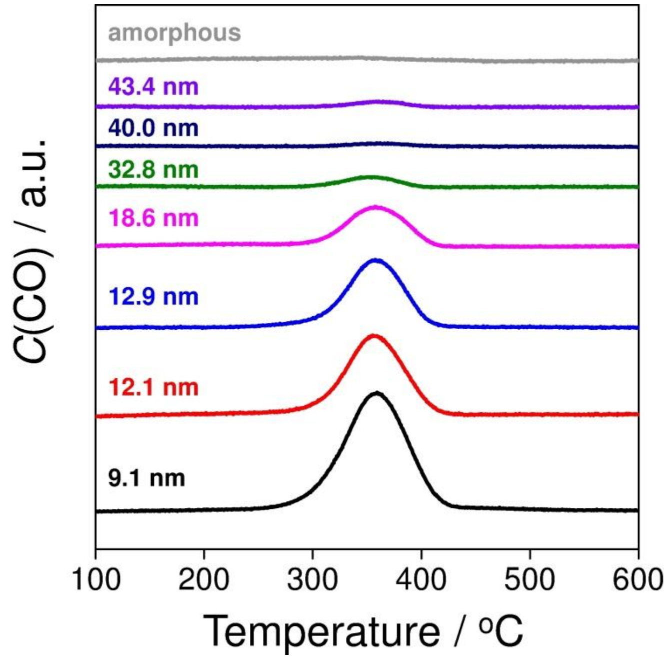

Supplementary Figure 13 CO-TPD profiles of selected ZrO_2 catalysts differing in their crystallite size.

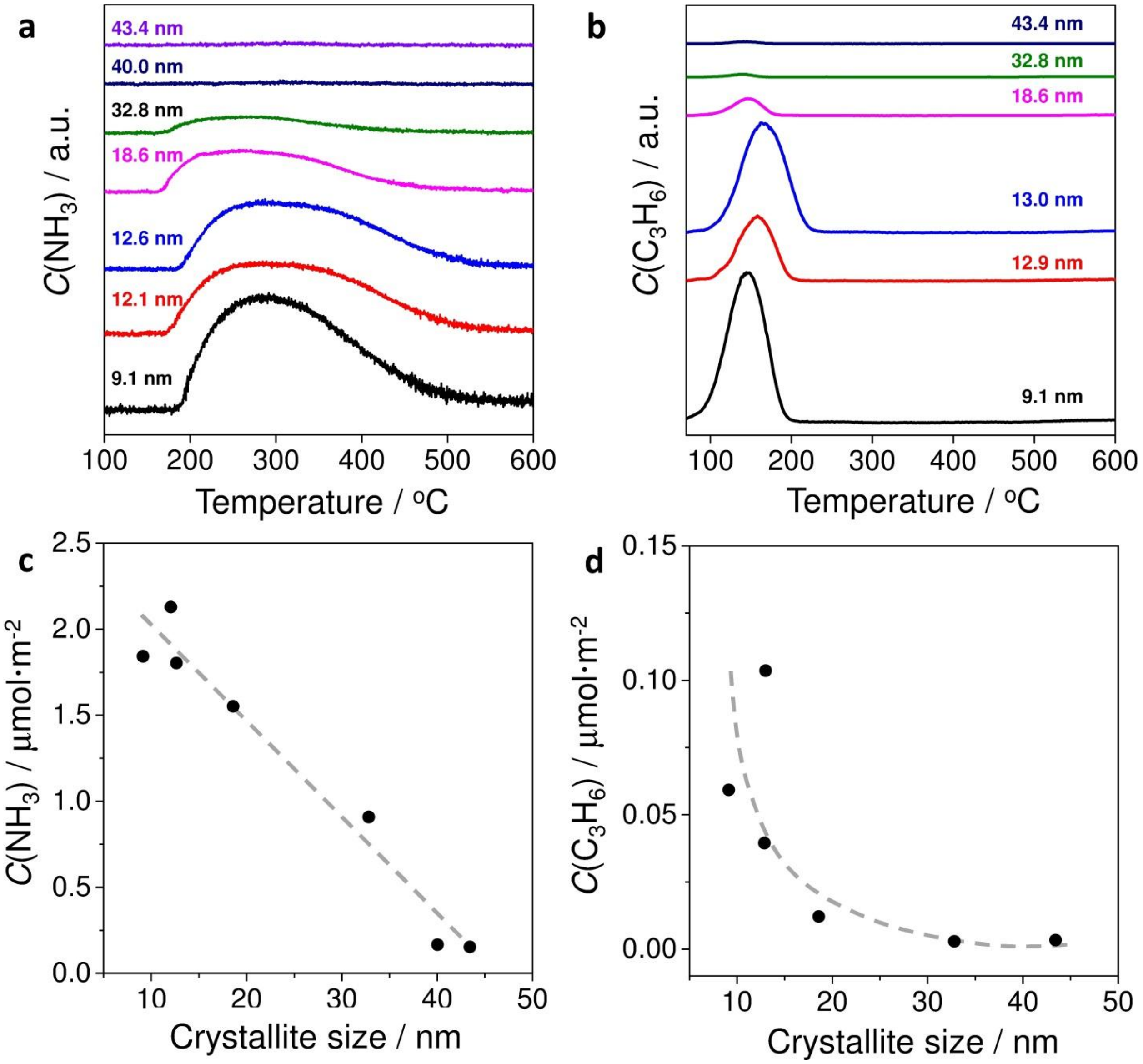

Supplementary Figure 14 TPD-profiles of desorbed **a** NH_3 or **b** C_3H_6 as well as the concentration of sites for **c** NH_3 or **d** C_3H_6 adsorption as determined from the NH_3 or C_3H_6 profiles of selected ZrO_2 catalysts differing in their crystallite size.

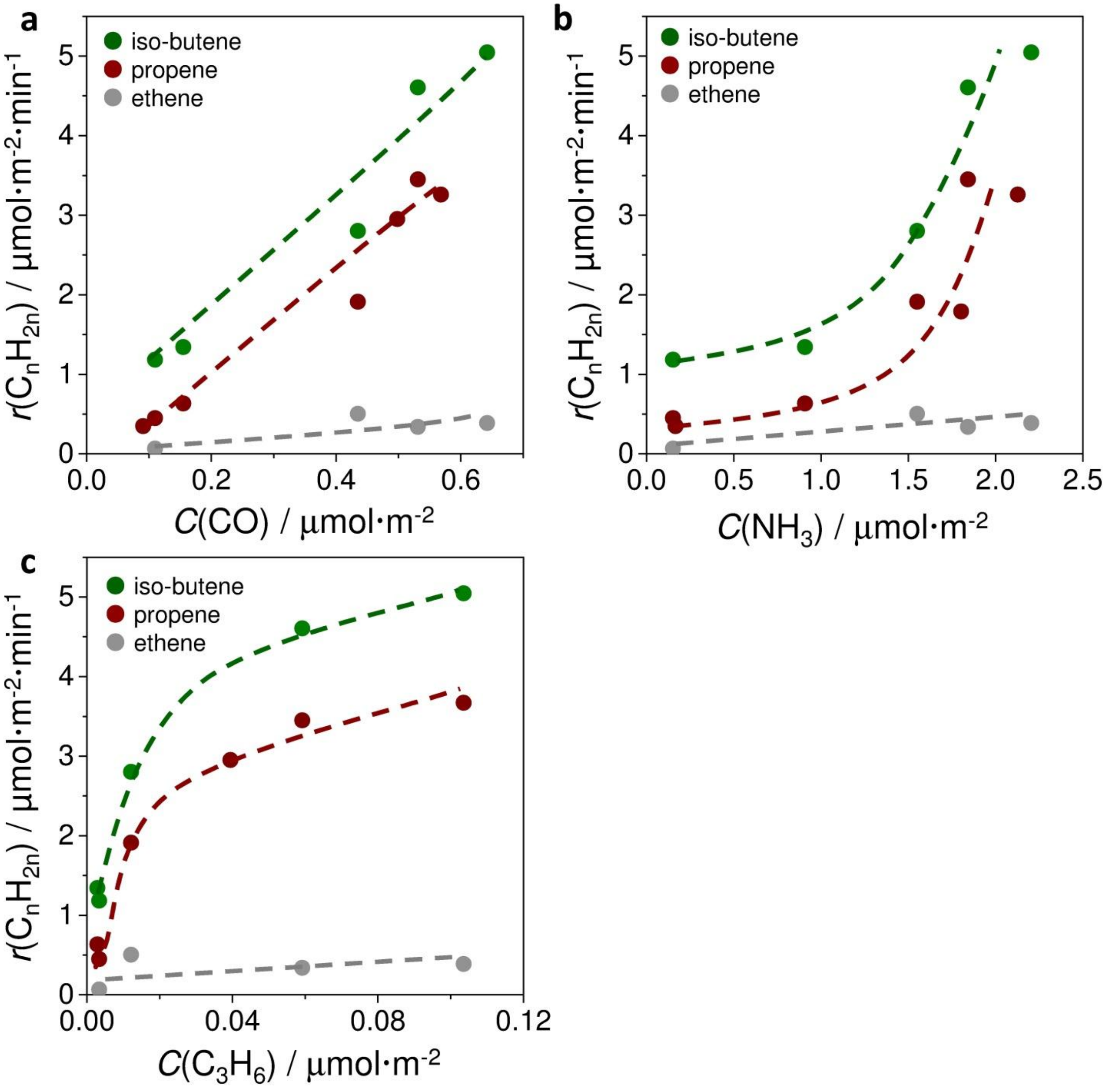

Supplementary Figure 15 The rate of ethene, propene or iso-butene formation upon ethane, propane or iso-butane dehydrogenation over ZrO_2 with differently sized crystallites versus the number of acidic sites determined from the amount of desorbed **a** CO, **b** NH_3 or **c** C_3H_6. Reaction conditions: T=550 °C, feed containing 40 vol% C_mH_{m+2} in N_2. *m*=2, 3, or 4.

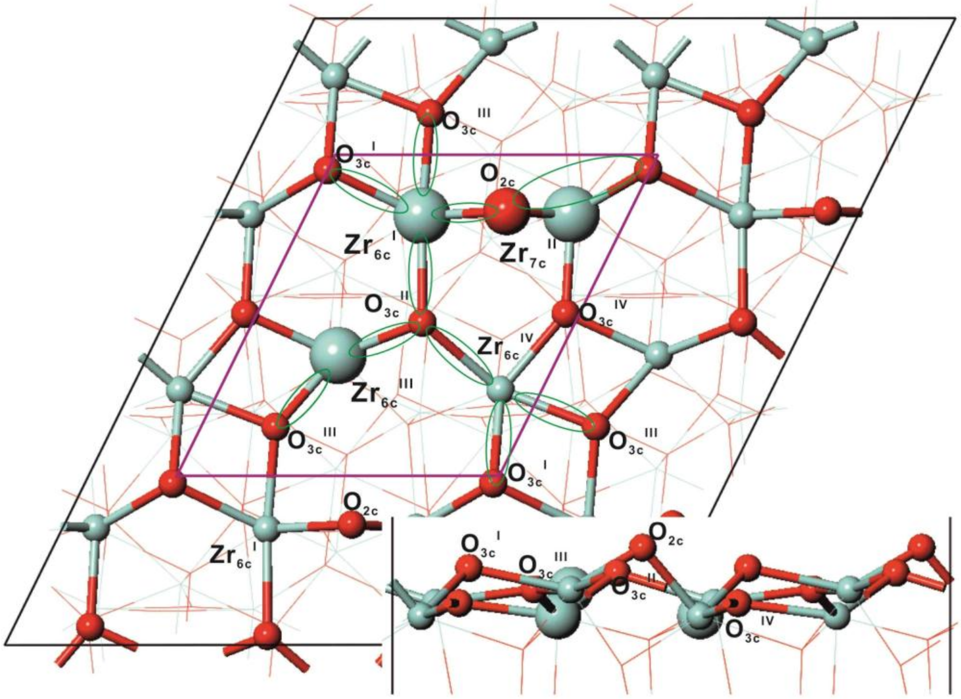

Supplementary Figure 16 Top view of the unit cell showing the possible adsorption sites of *m*-$ZrO_2(\bar{1}11)$ model (side view in the small inset at the lower-right corner) (Zr/cyan; O/red)

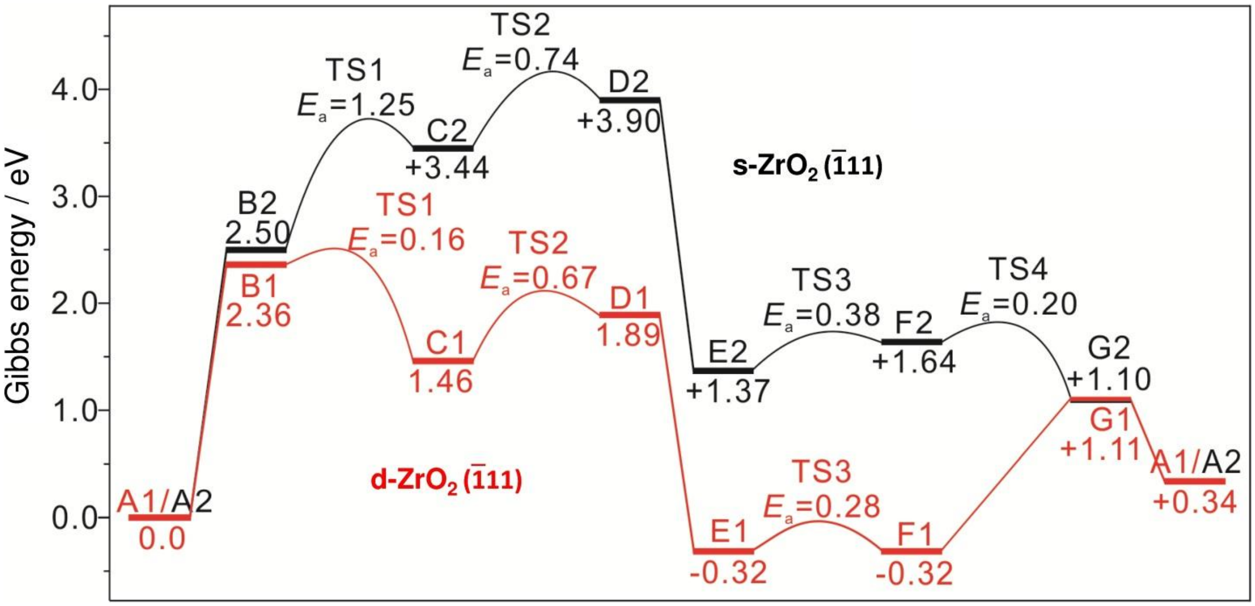

Supplementary Figure 17 Gibbs free energy pathways calculated for propane dehydrogenation to propene on the s-$ZrO_2(\bar{1}11)$ and (**c**) d-$ZrO_2(\bar{1}11)$ surfaces at 823.15 K and ambient pressure.

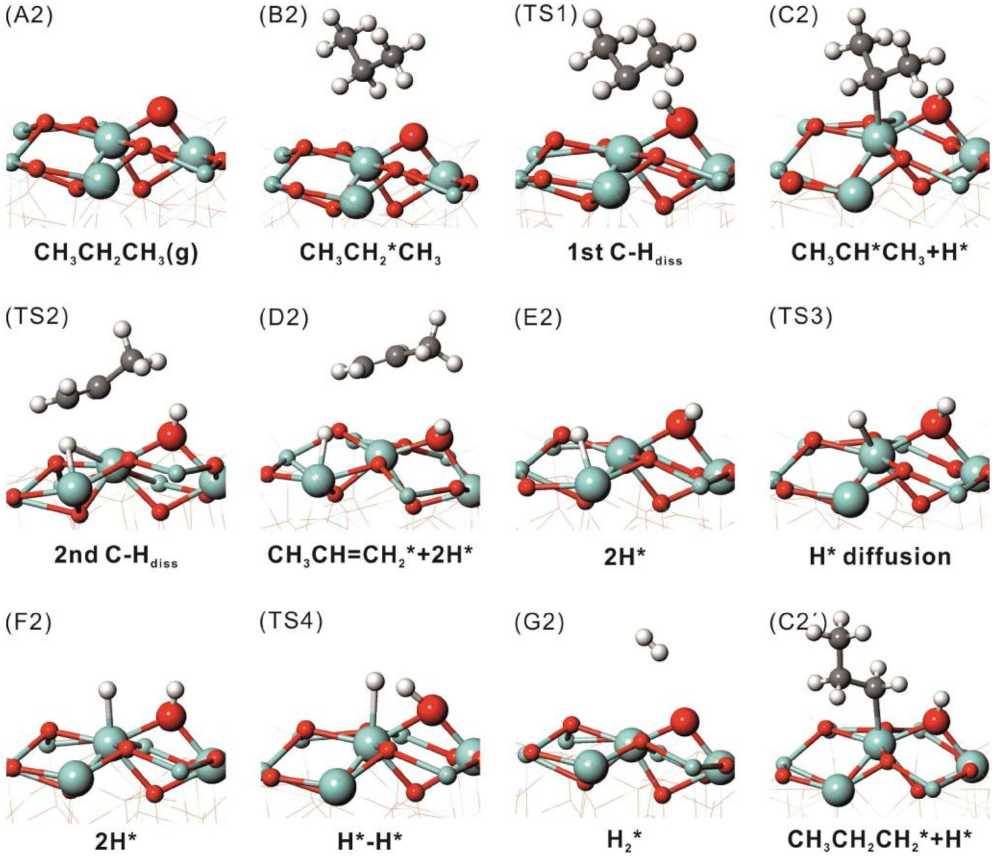

Supplementary Figure 18 Optimized structures of intermediates along the most preferred pathway of propane dehydrogenation to propene on the s-$ZrO_2(\bar{1}11)$ surface.

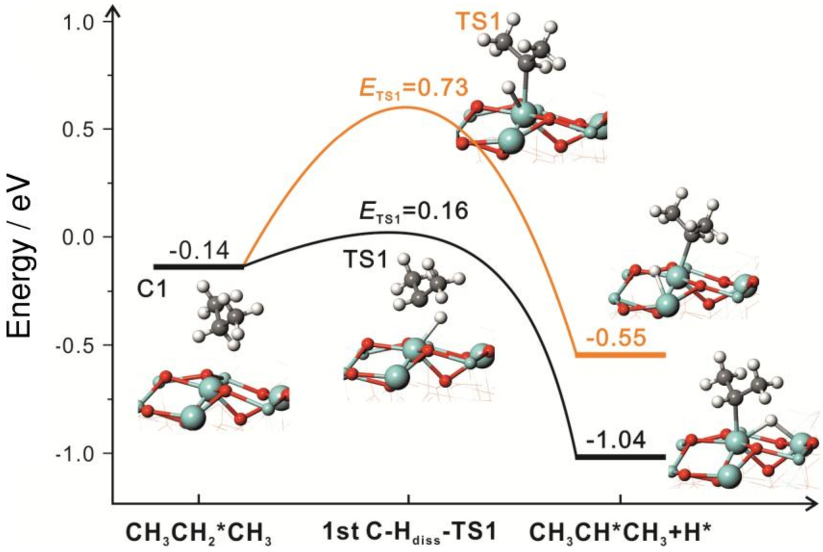

Supplementary Figure 19 Energy profiles of the first methylene C–H bond dissociation on the [Zr_{5c}^{I}, Zr_{6c}^{II}]-O_v (black) and [Zr_{5c}^{I}, Zr_{6c}^{III}]-O_v (orange) sites on the d-ZrO_2 ($\bar{1}$11) surface with the geometry at the adsorbed propane as well as the corresponding transition states and final states.

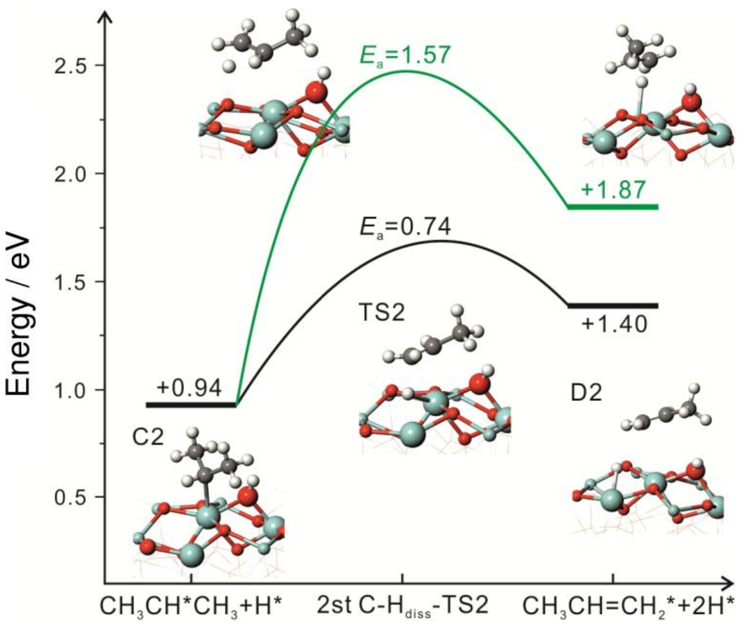

Supplementary Figure 20 Energy profiles of the methyl C–H bond dissociation on the Zr_{6c}^{I} and Zr_{6c}^{III} site (black) vs Zr_{6c}^{I} and Zr_{6c}^{IV} site (green) on s-ZrO_2 ($\bar{1}11$) surface with the geometry at the adsorbed isopropyl (C2) as well as the corresponding transition states (TS2) and final states (D2).

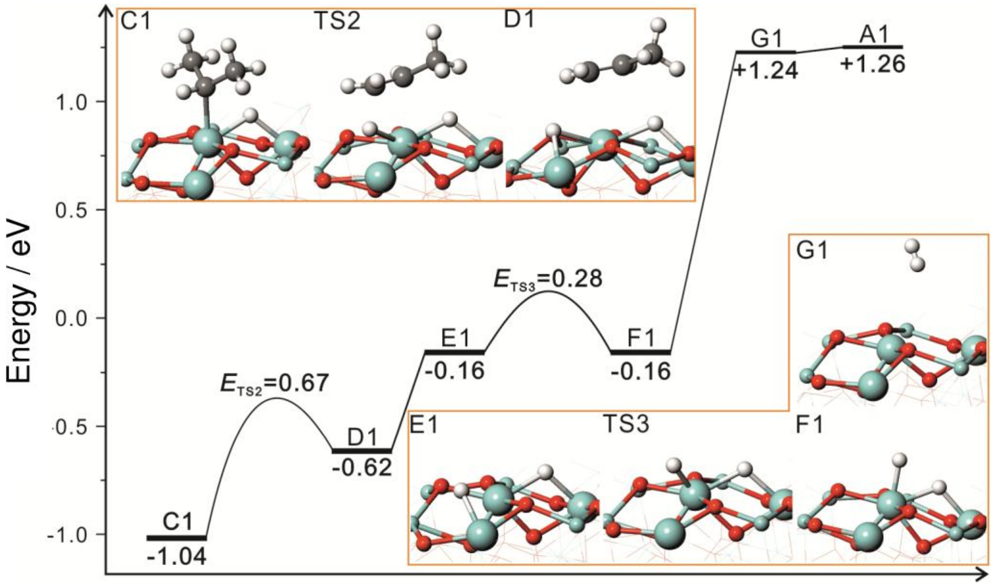

Supplementary Figure 21 Energy profiles and optimized structures of intermediates along the pathway of isopropyl dehydrogenation to propene on d-$ZrO_2(\bar{1}11)$.

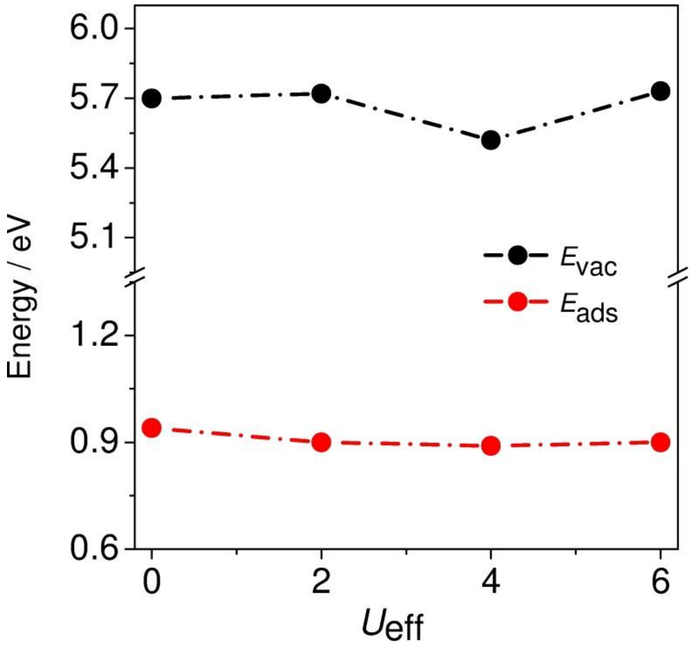

Supplementary Figure 22 Oxygen vacancy formation energy, E_{vac}, of the $ZrO_2(\bar{1}11)$ surface (black circle) and dissociative adsorption energy, E_{ads}, of C_3H_8 on $ZrO_2(\bar{1}11)$ (red circle) computed using the PBE+U functional, as a function of U_{eff}.

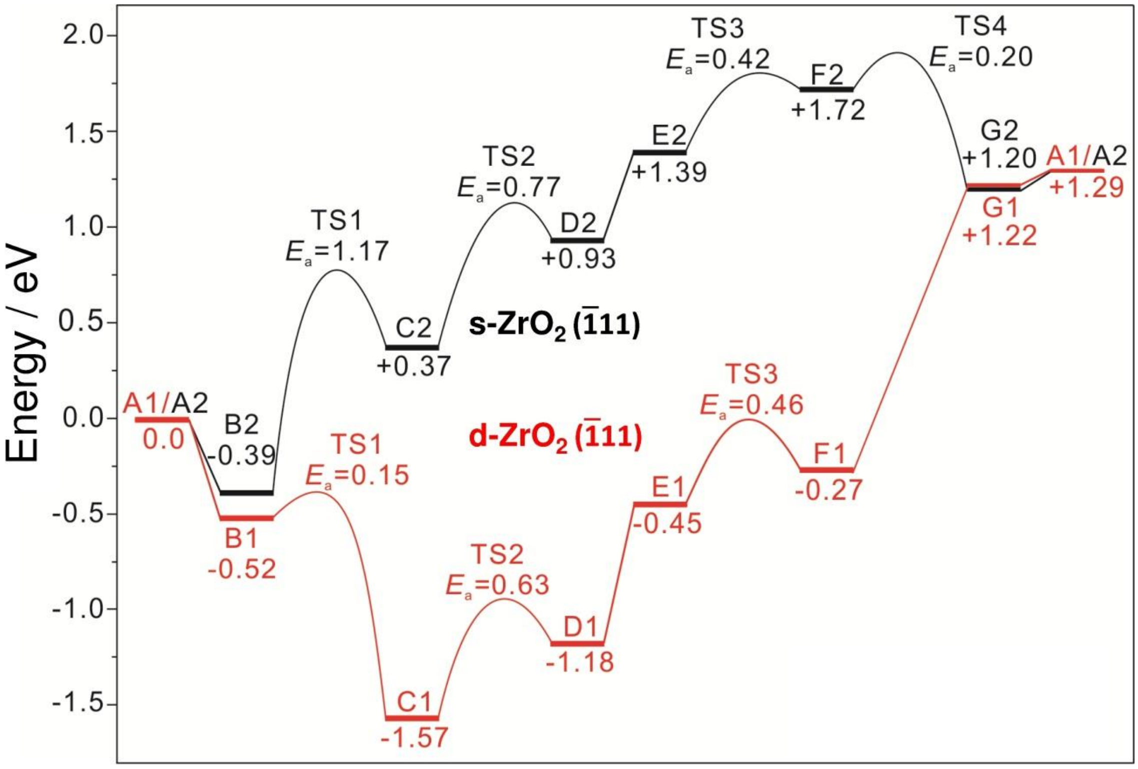

Supplementary Figure 23 DFT-D3 calculated energy profiles along the pathways of propane dehydrogenation to propene on s-$ZrO_2(\bar{1}11)$ and d-$ZrO_2(\bar{1}11)$ surfaces.

Supplementary Table 1 Preparation details and selected physical properties of all ZrO_2 catalysts.

Catalyst	Zirconia precursor	Preparation method	S_{BET} / $m^2 \cdot g^{-1}$	Crystallite size / nm
ZrO_2_1	$ZrO(NO_3)_2 \cdot xH_2O$	hydrothermal	116.2	7.3
ZrO_2_2	$ZrO(NO_3)_2 \cdot xH_2O$	hydrothermal	113.0	7.2
ZrO_2_3	$ZrO(NO_3)_2 \cdot xH_2O$	hydrothermal	123.2	7.1
ZrO_2_4	$ZrO(NO_3)_2 \cdot xH_2O$	hydrothermal	116.3	7.3
ZrO_2_5	$ZrO(NO_3)_2 \cdot xH_2O$	hydrothermal	100.9	7.7
ZrO_2_6	$ZrO(NO_3)_2 \cdot xH_2O$	hydrothermal	91.3	7.9
ZrO_2_7	$ZrO(NO_3)_2 \cdot xH_2O$	hydrothermal	77.2	9.3
ZrO_2_8	$ZrO(NO_3)_2 \cdot xH_2O$	hydrothermal	51.3	12.0
ZrO_2_9	$ZrO(NO_3)_2 \cdot xH_2O$	hydrothermal	49.6	12.4
ZrO_2_10	$ZrO(NO_3)_2 \cdot xH_2O$	hydrothermal	53.7	10.7
ZrO_2_11	$ZrO(NO_3)_2 \cdot xH_2O$	hydrothermal	52.7	12.1
ZrO_2_12	$ZrO(NO_3)_2 \cdot xH_2O$	hydrothermal	51.6	10.4
ZrO_2_13	$ZrO(NO_3)_2 \cdot xH_2O$	hydrothermal	53.2	12.7
ZrO_2_14	$ZrO(NO_3)_2 \cdot xH_2O$	hydrothermal	57.0	12.6
ZrO_2_15	$ZrO(NO_3)_2 \cdot xH_2O$	hydrothermal	51.2	10.5
ZrO_2_16	$ZrO(NO_3)_2 \cdot xH_2O$	hydrothermal	64.8	10.5
ZrO_2_17	$ZrO(NO_3)_2 \cdot xH_2O$	hydrothermal	49.4	11.2
ZrO_2_18	$ZrO(NO_3)_2 \cdot xH_2O$	hydrothermal	45.3	11.8
ZrO_2_19	$ZrO(NO_3)_2 \cdot xH_2O$	hydrothermal	53.6	11.9
ZrO_2_20	$ZrO(NO_3)_2 \cdot xH_2O$	hydrothermal	50.1	12.7
ZrO_2_21	$ZrO(NO_3)_2 \cdot xH_2O$	hydrothermal	38.9	12.8
ZrO_2_22	$ZrO(NO_3)_2 \cdot xH_2O$	hydrothermal	42.9	13.0
ZrO_2_23	$ZrO(NO_3)_2 \cdot xH_2O$	hydrothermal	21.4	21.5
ZrO_2_24	$ZrO(NO_3)_2 \cdot xH_2O$	precipitation	246.6	amorphous
ZrO_2_25	$ZrOCl_2 \cdot 8H_2O$	precipitation	46.8	14.4
ZrO_2_26	$ZrOCl_2 \cdot 8H_2O$	precipitation	267.3	amorphous
ZrO_2_27	$Zr(OC_4H_9)_4$	sol-gel	16.3	24.8
ZrO_2_28	$Zr(OC_4H_9)_4$	sol-gel	16.8	26.1
ZrO_2_29	$Zr(OC_4H_9)_4$	sol-gel	17.1	29.7
ZrO_2_30	$Zr(OC_4H_9)_4$	sol-gel	19.6	35.4
ZrO_2_31	$Zr(OC_3H_7)_4$	sol-gel	12.5	35.8
ZrO_2_32	$Zr(OC_3H_7)_4$	sol-gel	12.7	40.0
ZrO_2_33	$ZrO(NO_3)_2 \cdot xH_2O$	filter paper templating	25.2	31.9
ZrO_2_34	$ZrO(NO_3)_2 \cdot xH_2O$	calcination	17.7	43.4
ZrO_2_35	-	provided by Saint-Gobain	88.4	9.1
ZrO_2_36	-	calcination	54.0	12.9
ZrO_2_37	-	calcination	37.9	18.6
ZrO_2_38	-	calcination	23.3	32.8

Supplementary Table 2 ZrO_2 samples calcined at temperatures (T_{calc}) lower than 550°C, the size of crystallites and the specific surface area in their fresh form and after PDH at 550°C.

Catalysts	T_{calc} / °C	Fresh catalyst		Spent catalyst	
		S_{BET} / $m^2 \cdot g^{-1}$	Crystallite size / nm	S_{BET} / $m^2 \cdot g^{-1}$	Crystallite size / nm
ZrO_2_1	110	199.9	4.8	116.2	7.3
ZrO_2_2	200	175.7	5.0	113.0	7.2
ZrO_2_3	250	174.0	5.0	123.2	7.1
ZrO_2_4	300	175.4	5.0	116.3	7.3
ZrO_2_5	350	125.0	6.0	100.9	7.7
ZrO_2_6	400	102.2	6.7	91.3	7.9
ZrO_2_7	450	74.9	8.7	77.2	9.3

Supplementary Table 3 Formation energy of oxygen vacancy at various positions (E_f, eV) and reaction energies of H_2 and CO oxidation on m-$ZrO_2(\bar{1}11)$ surface respectively.

Site	E_f /eV	ΔE_r (H_2) /eV	ΔE_r (CO)/eV
O_{2c}	5.70	3.17	2.44
O_{3c}^{I}	5.96	3.43	2.70
O_{3c}^{II}	6.08	3.54	2.82
O_{3c}^{III}	5.90	3.37	2.64
O_{3c}^{IV}	6.04	3.51	2.78

Supplementary Table 4 Catalytic performance of various catalysts from literature and from the present study in the non-oxidative propane dehydrogenation to propene.

Catalyst	m / g	T / °C	$X(C_3H_6)$ / %	$Y(C_3H_6)$ / %	F_{total} / $mL \cdot min^{-1}$	Feeding gas composition	WHSV / h^{-1}	$STY(C_3H_6)$ /$kg \cdot h^{-1} \cdot kg^{-1}$	Ref.
$PtGaK/Al_2O_3$	0.15	620	41.9	40.6	9	pure C_3H_8	7.1	2.74	[24]
Pt/Na-ZSM-5	0.4	555	44.8	24.8		pure C_3H_8	2.6	0.62	[25]
$Pt–Sn/CeO_2$	0.1	680	39.5	33.4	12	C_3H_8/Carrier gas=1/5, traces of H_2O	2.4	0.75	[26]
PtSn/TS-1		590	53.5	49.4		$C_3H_8:H_2:N_2$ = 1:1:4	3.0	1.41	[27]
$Pt-Sn/\gamma-Al_2O_3$	0.3	600	38.2	35.5	9	pure C_3H_8	3.2	1.08	[28]
$PtCe/\gamma-Ga_xAl_{(2-x)}O_3$		620	60.0	58.8		C_3H_8/He=0.25	5.4	3.03	[29]
$PtSn/Al_2O_3$ sheet		590	48.7	48.3		16.2 vol% C_3H_8, 20.3 vol% H_2 in N_2	9.4	4.33	[30]
$Pt–Sn/MgAl_2O_4$	0.1	580	38.9	38.8	20	10 vol% C_3H_8, 10% H_2 in He	2.4	0.87	[31]
$Pt/TiO_2-Al_2O_3$	0.15	600	45.5	40.5	50	26 vol% C_3H_8, 26 vol% H_2 in N_2	10.0	3.87	[32]
Ga/Cr-α-ZrP	0.12	550	27.6	22.1	28.3	7.06 vol% C_3H_8 in He	2.0	0.41	[33]
Cr_2O_3/ZrO_2	0.2	550	60.9	46.4	20	2.5 vol% C_3H_8 in N_2	0.3	0.13	[34]
Cr5/SBA-1	0.2	550	33.0	28.4	30	$N_2:C_3H_8$:He = 5:1:9	1.2	0.32	[35]
$Cr_2O_3-K_2O/Al_2O_3$	0.3	550	42.1	36.7	10	40 vol% C_3H_8 in N_2	1.6	0.55	[36]
$Cr_2O_3-K_2O/Al_2O_3$	0.3	600	50.8	43.3	40	40 vol% C_3H_8 in N_2	6.3	2.59	[36]
$Cr_2O_3-K_2O/Al_2O_3$	0.3	625	42.1	35.3	60	40 vol% C_3H_8 in N_2	9.4	3.18	[36]
$Ru/YZrO_x$	0.3	550		30.0	10	40 vol% C_3H_8 in N_2	1.6	0.45	[36]
$Ru/YZrO_x$	0.3	600		40.0	40	40 vol% C_3H_8 in N_2	6.3	2.40	[36]
$Ru/YZrO_x$	0.3	625		35.0	60	40 vol% C_3H_8 in N_2	9.4	3.15	[36]
Ga_2O_3	0.5	600	33.0	30.7	30	17vol% C_3H_8 in CO_2	1.2	0.35	[37]
Ga_2O_3/Al_2O_3	0.2	600	33.0	30.4	20	2.5 vol% C_3H_8 in N_2	0.3	0.09	[38]
VO_x/Al_2O_3	0.25	600	32.0	30.1	25	28 vol% C_3H_8, 28% H_2 in N_2	3.0	0.86	[39]
ZrO_2(reduced in CO)	0.07	550	28.4	24.7	10	40 vol% C_3H_8 in N_2	6.7	1.58	This work
ZrO_2(reduced in CO)	0.08	600	28.3	24.2	20	40 vol% C_3H_8 in N_2	11.7	2.71	This work
ZrO_2(reduced in CO)	0.1	625	27.8	23.4	30	40 vol% C_3H_8 in N_2	14.1	3.15	This work

Supplementary Table 5 Total electrical conductivity of selected ZrO_2 catalysts at 550 °C and different oxygen partial pressures.

Catalyst	Crystallite size / nm	Conductivity ($\sigma \times 10^9$ / $\Omega^{-1}\cdot mm^{-1}$)	
		20 kPa O_2	10^{-4} kPa O_2
ZrO_2_35	9.1	11.5	9.5
ZrO_2_36	12.9	8.3	5.8
ZrO_2_25	14.4	4.8	3.7
ZrO_2_37	18.6	6.6	5.3
ZrO_2_38	32.8	3.0	2.8
ZrO_2_32	40.0	2.3	1.9
ZrO_2_34	43.4	2.2	2.0
ZrO_2_26	amorphous	0.5	0.5

Supplementary Table 6 Comparison of calculated and experimentally determined bulk unit cell parameters for *m*-ZrO_2.

		Exp.[16]	Calc.[17]	Calc.[18]	Calc.[19]	Calc.[20]	Calc.[21]
a (Å)	5.144	5.151	5.192	5.211	5.184	5.209	5.198
b (Å)	5.262	5.212	5.265	5.286	5.274	5.280	5.280
c (Å)	5.285	5.317	5.358	5.388	5.358	5.391	5.350
β (deg)	99.36	99.22	99.81	99.59	99.30	99.58	99.53

Supplementary Table 7 Methylene C-H bond dissociative adsorption energy (E_{ads}, eV) of *iso*-C_3H_7 and H at different sites on s-$ZrO_2(\bar{1}11)$.

Adsorption sites	*iso*-C_3H_7	H	E_{ads}
Zr_{6c}^{I}-O_{2c}	Zr_{6c}^{I}	O_{2c}	0.94
	O_{2c}	Zr_{6c}^{I}	1.39
Zr_{6c}^{I}-O_{3c}^{I}	Zr_{6c}^{I}	O_{3c}^{I}	1.81
Zr_{6c}^{I}-O_{3c}^{II}	Zr_{6c}^{I}	O_{3c}^{II}	1.53
Zr_{6c}^{I}-O_{3c}^{III}	Zr_{6c}^{I}	O_{3c}^{III}	1.77
Zr_{6c}^{III}-O_{3c}^{II}	Zr_{6c}^{III}	O_{3c}^{II}	1.46
Zr_{6c}^{III}-O_{3c}^{III}	Zr_{6c}^{III}	O_{3c}^{III}	1.70
Zr_{6c}^{IV}-O_{3c}^{I}	Zr_{6c}^{IV}	O_{3c}^{I}	1.69
Zr_{6c}^{IV}-O_{3c}^{II}	Zr_{6c}^{IV}	O_{3c}^{II}	1.61
Zr_{6c}^{IV}-O_{3c}^{III}	Zr_{6c}^{IV}	O_{3c}^{III}	1.70
O_{2c}-O_{3c}^{I}	O_{2c}	O_{3c}^{I}	3.34
	O_{3c}^{I}	O_{2c}	3.21
Zr_{6c}^{I}-O_{2c} (methyl)	Zr_{6c}^{I}	O_{2c}	0.86

Supplementary Table 8 Methylene C-H bond dissociative adsorption energy (E_{ads}, eV) of *iso*-C_3H_7 and H at different sites on d-$ZrO_2(\bar{1}11)$.

Adsorption sites	*iso*-C_3H_7	H	E_{ads}
[Zr_{5c}^{I}, Zr_{6c}^{II}]-O_v	Zr_{5c}^{I}	Bridge of Zr_{5c}^{I} and Zr_{6c}^{II}	-1.04
[Zr_{5c}^{I}, Zr_{6c}^{III}]-O_v	Zr_{5c}^{I}	Bridge of Zr_{5c}^{I} and Zr_{6c}^{III}	-0.55
Zr_{5c}^{I}-O_{3c}^{I}	Zr_{5c}^{I}	O_{3c}^{I}	1.38
Zr_{5c}^{I}-O_{3c}^{II}	Zr_{5c}^{I}	O_{3c}^{II}	1.27
Zr_{5c}^{I}-O_{3c}^{III}	Zr_{5c}^{I}	O_{3c}^{III}	1.34
Zr_{6c}^{III}-O_{3c}^{II}	Zr_{6c}^{III}	O_{3c}^{II}	1.40
Zr_{6c}^{III}-O_{3c}^{III}	Zr_{6c}^{III}	O_{3c}^{III}	1.65
Zr_{6c}^{IV}-O_{3c}^{I}	$Zr_{6c}^{IV}Zr^{IV}$	O_{3c}^{I}	1.61
Zr_{6c}^{IV}-O_{3c}^{II}	Zr_{6c}^{IV}	O_{3c}^{II}	1.58
Zr_{6c}^{IV}-O_{3c}^{III}	Zr_{6c}^{IV}	O_{3c}^{III}	1.73

Supplementary References

1. Li, W. et al. Facile Synthesis of Pure Monoclinic and Tetragonal Zirconia Nanoparticles and Their Phase Effects on the Behavior of Supported Molybdena Catalysts for Methanol-Selective Oxidation. *Langmuir* **24**, 8358-8366 (2008).
2. Shigapov, A. N., Graham, G. W., McCabe, R. W. & Plummer, H. K. The preparation of high-surface area, thermally-stable, metal-oxide catalysts and supports by a cellulose templating approach. *Appl. Catal. A* **210**, 287-300 (2001).
3. Kresse, G. & Furthmüller, J. Efficiency of ab-initio total energy calculations for metals and semiconductors using a plane-wave basis set. *Comput. Mater. Sci.* **6**, 15-50 (1996).
4. Kresse, G. & Furthmüller, J. Efficient iterative schemes for ab initio total-energy calculations using a plane-wave basis set. *Phys. Rev. B* **54**, 11169-11186 (1996).
5. Perdew, J. P., Burke, K. & Ernzerhof, M. Generalized Gradient Approximation Made Simple. *Phys. Rev. Lett.* **77**, 3865-3868 (1996).
6. Henkelman, G., Uberuaga, B. P. & Jónsson, H. A climbing image nudged elastic band method for finding saddle points and minimum energy paths. *J. Chem. Phys.* **113**, 9901-9904 (2000).
7. Monkhorst, H. J. & Pack, J. D. Special points for Brillouin-zone integrations. *Phys. Rev. B* **13**, 5188-5192 (1976).
8. Ganduglia-Pirovano, M. V., Hofmann, A. & Sauer, J. Oxygen vacancies in transition metal and rare earth oxides: Current state of understanding and remaining challenges. *Surf. Sci. Rep.* **62**, 219-270 (2007).
9. Syzgantseva, O. A., Calatayud, M. & Minot, C. Revealing the Surface Reactivity of Zirconia by Periodic DFT Calculations. *J. Phys. Chem. C* **116**, 6636-6644 (2012).
10. Dudarev, S. L. et al. Electron-energy-loss spectra and the structural stability of nickel oxide: An LSDA+U study. *Phys. Rev. B* **57**, 1505-1509 (1998).
11. Grimme, S., Antony, J., Ehrlich, S. & Krieg, H. A consistent and accurate ab initio parametrization of density functional dispersion correction (DFT-D) for the 94 elements H-Pu. *J. Chem. Phys.* **132**, 154104 (2010).
12. Weaver, J. F., Hakanoglu, C., Antony, A. & Asthagiri, A. Alkane activation on crystalline metal oxide surfaces. *Chem. Soc. Rev.* **43**, 7536-7547 (2014).
13. Li, T. et al. Adsorption of alkanes on stoichiometric and oxygen-rich RuO2(110). *Phys. Chem. Chem. Phy.* **18**, 22647-22660 (2016).
14. Bian, Y. et al. Facile Dehydrogenation of Ethane on the IrO_2(110) Surface. *J. Am. Chem. Soc.* **140**, 2665-2672 (2018).
15. Liang, Z. et al. Low-temperature activation of methane on the IrO_2 (110) surface. *Science* **356**, 299-303 (2017).
16. Howard, C. J., Hill, R. J. & Reichert, B. E. Structures of ZrO_2 polymorphs at room temperature by high-resolution neutron powder diffraction. *Acta Cryst. Sect. B* **44**, 116-120 (1988).
17. Foster, A. S. et al. Structure and electrical levels of point defects in monoclinic zirconia. *Phys. Rev. B* **64**, 224108 (2001).
18. Kuwabara, A., Tohei, T., Yamamoto, T. & Tanaka, I. Ab initio lattice dynamics and phase transformations of ZrO_2. *Phys. Rev. B* **71**, 064301 (2005).
19. Korhonen, S. T., Calatayud, M. & Krause, A. O. I. Stability of Hydroxylated $(\bar{1}11)$ and $(\bar{1}01)$ Surfaces of Monoclinic Zirconia: A Combined Study by DFT and Infrared Spectroscopy. *J. Phys. Chem. C* **112**, 6469-6476 (2008).

20. Wu, H. et al. First-principles study of phase transition and band structure of ZrO_2 under pressure. *J. Alloys and Comp.* **645**, 352-357 (2015).
21. Liang, Z. et al. Structural, mechanical and thermodynamic properties of ZrO_2 polymorphs by first-principles calculation. *Physica B: Condensed Matter* **511**, 10-19 (2017).
22. Christensen, A. & Carter, E. A. First-principles study of the surfaces of zirconia. *Phys. Rev. B* **58**, 8050-8064 (1998).
23. Piskorz, W. et al. Periodic DFT and Atomistic Thermodynamic Modeling of the Surface Hydration Equilibria and Morphology of Monoclinic ZrO_2 Nanocrystals. *J. Phys. Chem. C* **115**, 24274-24286 (2011).
24. Sattler, J. J. H. B. et al. Platinum-promoted g-/Al_2O_3 as highly active, selective, and stable catalyst for the dehydrogenation of propane. *Angew. Chem., Int. Ed.* **53**, 9251-9256 (2014).
25. De Cola, P. L., Gläser, R. & Weitkamp, J. Non-oxidative propane dehydrogenation over Pt–Zn-containing zeolites. *Appl. Catal. A* **306**, 85-97 (2006).
26. Xiong, H. et al. Thermally Stable and Regenerable Platinum–Tin Clusters for Propane Dehydrogenation Prepared by Atom Trapping on Ceria. *Angew. Chem., Int. Ed.* **56**, 8986-8991 (2017).
27. Li, J. et al. Size effect of TS-1 supports on the catalytic performance of PtSn/TS-1 catalysts for propane dehydrogenation. *J. Catal.* **352**, 361-370 (2017).
28. Pham, H. N., Sattler, J. J. H. B., Weckhuysen, B. M. & Datye, A. K. Role of Sn in the Regeneration of Pt/γ-Al_2O_3 Light Alkane Dehydrogenation Catalysts. *ACS Catal.* **6**, 2257-2264 (2016).
29. Im, J. & Choi, M. Physicochemical Stabilization of Pt against Sintering for a Dehydrogenation Catalyst with High Activity, Selectivity, and Durability. *ACS Catal.* **6**, 2819-2826 (2016).
30. Shi, L. et al. Al_2O_3 Nanosheets Rich in Pentacoordinate Al^{3+} Ions Stabilize Pt-Sn Clusters for Propane Dehydrogenation. *Angew. Chem., Int. Ed.* **54**, 13994-13998 (2015).
31. Zhu, H. et al. Sn surface-enriched Pt–Sn bimetallic nanoparticles as a selective and stable catalyst for propane dehydrogenation. *J. Catal.* **320**, 52-62 (2014).
32. Jiang, F. et al. Propane Dehydrogenation over Pt/TiO_2–Al_2O_3 Catalysts. *ACS Catal.* **5**, 438-447 (2015).
33. Alcántara-Rodríguez, M., Rodríguez-Castellón, E. & Jiménez-López, A. Propane Dehydrogenation on Mixed Ga/Cr Oxide Pillared Zirconium Phosphate Materials. *Langmuir* **15**, 1115-1120 (1999).
34. Zhang, X., Yue, Y. & Gao, Z. Chromium Oxide Supported on Mesoporous SBA-15 as Propane Dehydrogenation and Oxidative Dehydrogenation Catalysts. *Catal. Lett.* **83**, 19-25 (2002).
35. Michorczyk, P., Pietrzyk, P. & Ogonowski, J. Preparation and characterization of SBA-1–supported chromium oxide catalysts for CO_2 assisted dehydrogenation of propane. *Micropor. Mesopor. Mater.* **161**, 56-66 (2012).
36. Otroshchenko, T. et al. ZrO_2-based unconventional catalysts for non-oxidative propane dehydrogenation: Factors determining catalytic activity. *J. Catal.* **348**, 282-290 (2017).
37. Michorczyk, P. & Ogonowski, J. Dehydrogenation of propane to propene over gallium oxide in the presence of CO_2. *Appl. Catal. A* **251**, 425-433 (2003).
38. Xu, B. J. et al. Support effect in dehydrogenation of propane in the presence of CO_2 over supported gallium oxide catalysts. *J. Catal.* **239**, 470-477 (2006).

39. Liu, G. et al. Nature of the Active Sites of VO_x/Al_2O_3 Catalysts for Propane Dehydrogenation. *ACS Catal.* **6**, 5207-5214 (2016).
